# Intermolecular Nuclear Spin Hyperpolarization Transfer via Cross‐Relaxation Triggers RASER of Solute Molecules

**DOI:** 10.1002/anie.2865398

**Published:** 2026-03-26

**Authors:** Ivan A. Trofimov, Anna P. Yi, Oleg G. Salnikov, Andrey N. Pravdivtsev, Henri de Maissin, Eduard Y. Chekmenev, Jan‐Bernd Hövener, Andreas B. Schmidt, Igor V. Koptyug

**Affiliations:** ^1^ Division of Medical Physics Department of Diagnostic and Interventional Radiology University Medical Center Freiburg Faculty of Medicine University of Freiburg Freiburg Germany; ^2^ International Tomography Center SB RAS Novosibirsk Russia; ^3^ Department of Natural Sciences Novosibirsk State University Novosibirsk Russia; ^4^ Section Biomedical Imaging Molecular Imaging North Competence Center (MOIN CC) Department of Radiology and Neuroradiology University Hospital Schleswig‐Holstein Kiel University Kiel Germany; ^5^ German Cancer Consortium (DKTK), Partner Site Freiburg a Partnership Between DKFZ and University Medical Center Freiburg Heidelberg Germany; ^6^ Department of Chemistry Integrative Biosciences (Ibio) Karmanos Cancer Institute (KCI) Wayne State University Detroit Michigan USA

**Keywords:** NMR spectroscopy, parahydrogen, RASER, NOE, hyperpolarization

## Abstract

Radiofrequency amplification by stimulated emission of radiation (RASER) is a phenomenon that is observed in systems of nuclear spins with strong inverse polarization beyond the thermal equilibrium. RASER is of fundamental interest and also possesses several features of possible practical importance, such as very narrow NMR lines and background‐free detection. However, so far, the effect was limited to molecules directly polarized by corresponding hyperpolarization techniques. In this work, we found that strongly hyperpolarized allylic compounds transferred polarization via nuclear Overhauser effect (NOE) to other solutes, triggering their RASER. In stark contrast to previous observations, the parahydrogen addition and intermolecular NOE transfer engendered RASER (PAINTER) does not require direct hyperpolarization of the target molecule. This way polarized and detected solutes had 10–20 times narrower lines compared to their classical NMR spectra, providing a useful analysis tool for various molecules beyond standard NMR limitations.

## Graphical abstract

Radiofrequency amplification by stimulated emission of radiation (RASER) of solutes was achieved via parahydrogen‐induced polarization (PHIP) and polarization transfer from the produced hyperpolarization donors to solutes via the intermolecular nuclear Overhauser effect (NOE). This paves the way toward high‐resolution mixture analysis as RASER signals are much better spectrally resolved than conventional NMR signals.

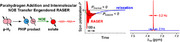

## 1 Introduction

Low sensitivity is a cornerstone issue in the field of nuclear magnetic resonance (NMR) spectroscopy. To overcome this, one can use hyperpolarization techniques that bring nuclear spin polarization far beyond the thermal equilibrium. It is no exaggeration to say that the potential to increase the power of nuclear magnetic resonance by tapping into the vast reservoir of polarization, while not limitless, is huge. Hyperpolarizing nuclear spins has shown to increase the signal in magnetic resonance imaging by about 5 orders of magnitude, and 4 orders of magnitude in NMR [1, 2]. Prominent hyperpolarization techniques are dynamic nuclear polarization (DNP) [3, 4, 5], spin‐exchange optical pumping (SEOP) [6, 7, 8], parahydrogen‐induced polarization (PHIP) [9, 10, 11], and signal amplification by reversible exchange (SABRE) [12, 13, 14]. DNP and SEOP yield high polarization levels together with sufficient purity and biocompatibility for in vivo applications in humans [8, 15, 16, 17].

PHIP and SABRE are two rapidly developing technologies for hyperpolarization of selected molecules in the liquid state that utilize parahydrogen (*p*‐H_2_, the singlet nuclear spin state of molecular hydrogen) as a source of spin alignment. Both methods have multiple applications in catalysis [18, 19, 20] and were recently translated into biochemical applications [21, 22] including in vivo animal studies [23, 24, 25]. Compared to SEOP and DNP, these methods do not require superconducting magnets or cryogenics and are very fast (seconds) [26, 27, 28]. More specifically, PHIP is based on the pairwise addition of *p*‐H_2_ to an unsaturated precursor [29], followed by a spin order transfer to the desired nucleus [30, 31, 32] or direct detection. If the added protons in the product molecule are magnetically inequivalent, this results in hyperpolarization detectable after application of an appropriate radiofrequency (RF) pulse. This way, in parahydrogen and synthesis allow dramatically enhanced nuclear alignment (PASADENA) experiments, the addition of *p*‐H_2_ is performed at high‐field conditions [9, 10]. On the other hand, in adiabatic longitudinal transport after dissociation engenders net alignment (ALTADENA) experiments, PHIP is conducted at a low magnetic field with subsequent adiabatic sample transfer to the detection field [11]. Positive and negative spin polarization produced in these experiments results in absorptive and emissive NMR lines. These techniques allow quick generation of significant ^1^H polarization [33, 34], which may then be efficiently transferred to heteronuclei (e.g., ^13^C [32, 35, 36], ^15^N [37, 38, 39], or even quadrupolar nuclei like ^2^H [40] and ^14^N [41].) However, the requirement of an unsaturated precursor greatly limits the range of compounds that can be hyperpolarized by PHIP. The PHIP‐X method further increases this range to molecules that participate in proton exchange with the PHIP‐hyperpolarized molecules [42], although the efficiency of this approach needs to be improved for practical applications.

A broader mechanism of intermolecular polarization transfer is driven by nuclear‐nuclear dipolar interactions; this mechanism is also referred to as nuclear Overhauser effect (NOE). There is a vast array of studies involving intermolecular hyperpolarization transfer in solution via NOE, based on dissolution DNP [43, 44], SEOP [45, 46], optically induced triplet DNP [47], and SABRE [48, 49]. However, PHIP‐hyperpolarized molecules cannot serve as polarization sources for intermolecular NOE directly after hydrogenation, as *p*‐H_2_‐derived spin orders in both PASADENA and ALTADENA yield zero net magnetization. However, by applying RF pulses, one can create a total net magnetization [50, 51]. The same effect could be achieved spontaneously, once sufficiently strong negative polarization of one of the spins is created, and the system is positioned in a well‐tuned resonator with a high Q‐factor. The interaction between negatively polarized spins and a resonator can lead to spontaneous radiation damping while producing NMR emissions in parallel, a phenomenon known as radio amplification by stimulated emission of radiation (RASER). It was shown to occur in various hyperpolarization experiments [52, 53, 54, 55, 56, 57, 58, 59]. For RASER to occur, radiation damping rate 1/τ_*RD*_ (Equation 1) must be positive and must exceed the combined apparent transverse relaxation $1/T_{2}^{*}$ and pumping 1/τ_*p*_ rates (Equation 2):

(1) $$\begin{array}{c} \;1/\tau_{\mathrm{RD}}=-\left(\mu_{0}/4\right)\eta Q\gamma^{2}\hbar n_{S}P\; \end{array}$$

(2) $$\begin{array}{c} 1/\tau_{\mathrm{RD}}>1/T_{2}^{*}+1/\tau_{p}\; \end{array}$$

where μ_0_ is vacuum permeability, η is the coil filling factor, *Q* is the resonator quality factor, γ is the gyromagnetic ratio, ℏ is the reduced Planck constant, *n*_S_ is spin density, and *P* is the initial nuclear spin polarization. From these restrictions, it may be understood that RASER takes place only if spin polarization *P* < 0 (as only this way the radiation damping rate is positive); higher *Q* of the NMR coil and higher concentration *n*_S_ of hyperpolarized nuclei make RASER easier to achieve. A thorough description of the RASER phenomenon can be found elsewhere [58, 59]. Importantly, the RASER emissions may continue as long as polarization is sufficiently replenished, making *p*‐H_2_‐based methods well suited for RASER as it is easy to supply the source of spin order (*p*‐H_2_) continuously [56, 57]. If Equation 2 is satisfied, RASER emissions may occur spontaneously or can be triggered by an RF pulse [56].

The RASER effect is related to radiation damping, which affects NMR signals, causing broadening of absorptive signals and narrowing and suppressing emissive ones (e.g., negatively polarized spins in PHIP products) [55]. The latter fact makes total magnetization of the sample highly non‐equilibrium and positive, rendering intermolecular hyperpolarization transfer via NOE efficient [60, 61]. This effect, named parahydrogen and RASER‐induced NOE (PRINOE) [60], has been achieved in both PASADENA and ALTADENA experiments, allowing for the hyperpolarization of various nuclei (so far – ^1^H, ^19^F, and ^31^P) in a wide range of solutes [61]. However, RASER of these solutes was not achieved.

In this work, we harness recently reported strong RASER of allyl alcohol (and other allylic molecules) produced via PHIP [62] to induce RASER activity of the solutes via NOE, implementing PHIP–RASER–NOE–RASER sequence. The RASER NMR signals of the solutes were 10–20 times narrower than conventional NMR signals under otherwise the same conditions. We investigated this parahydrogen addition and intermolecular NOE transfer engendered RASER (PAINTER) effect with different HP donors and solutes and corroborated the results by numerical simulations.

## 2 Results and Discussion

Earlier, we demonstrated that PHIP‐hyperpolarized allylic compounds produce strong and long‐lasting RASER [62], indicating that they may also be promising polarization donors in PRINOE experiments. Hence, we used HP allyl alcohol (**1**) as a polarization donor, which was obtained via *p*‐H_2_ addition to propargyl alcohol (**1′**) over [Rh(dppb)(nbd)]BF_4_ catalyst (further referred to as **[Rh]**, where dppb — 1,4‐bis(diphenylphosphino)butane, and nbd — norbornadiene). While PRINOE allows the transfer of polarization to a wide range of compounds [61], we utilized benzene as a model solute because (*i*) it contains six magnetically equivalent protons with long *T*_1_ [61], which is beneficial for a strong PRINOE signal, and (*ii*) its NMR signal does not overlap with those of hyperpolarized allyl alcohol. Both benzene and **1′** were used in high concentrations of 0.8 M to ease the emergence of RASER and NMR signal observation.

**FIGURE 1:**
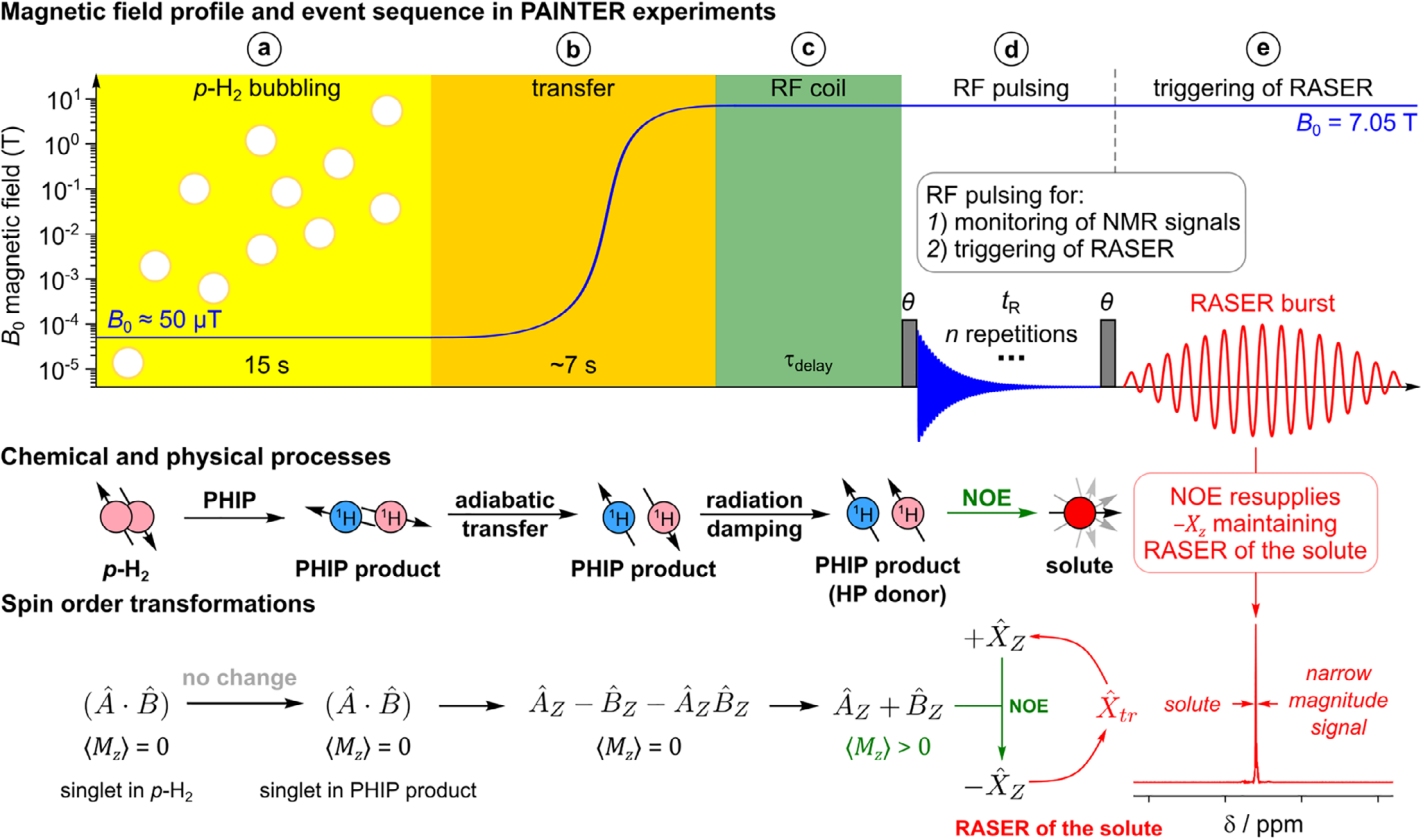
The schematic outline of *p*‐H_2_ addition and intermolecular NOE transfer engendered RASER (PAINTER) of the solutes, showing the experimental event sequence along with chemical and physical processes and spin order transformations. The PAINTER is achieved by the following sequence of experimental steps: (a) 15 s long *p*‐H_2_ bubbling at the Earth's magnetic field results in pairwise addition of *p*‐H_2_ to the C≡C‐bond of unsaturated precursor with formation of large amounts of HP product (*p*‐H_2_‐nascent spins denoted as *A* and *B*). The state is represented by a scalar product of two spins, $\left(\hat{A}\cdot \hat{B}\right)$, ignoring a unitary matrix and coefficients. The state is well retained upon hydrogenation. (b) Adiabatic transfer of the sample into the high field (7.05 T) converts *p*‐H_2_ spin order into longitudinal magnetizations of spins *A* and *B* of opposite signs. (c) Introduction of the sample into the RF coil converts negative longitudinal component $-{\hat{B}}_{z}$ into positive $+{\hat{B}}_{z}$ due to radiation damping and destroys in parallel the two spin order ${\hat{A}}_{z}{\hat{B}}_{z}$. The intermolecular cross‐relaxation starts transferring the resulting positive magnetization, to the solute molecules (represented by spin *X*), which leads to the buildup of their negative magnetization, $-{\hat{X}}_{z}$. (d) These processes can be optionally monitored by acquiring a series of NMR spectra with a flip angle *θ* and a repetition time *t*_R_. (e) Application of an RF pulse at the time point when $-{\hat{X}}_{z}$ exceeds the RASER threshold converts it to transversal plane, ${\hat{X}}_{tr}$, triggering RASER of the solute and flip of the corresponding spin order to $+{\hat{X}}_{z}$. For simplicity, the spin order expressions are provided without the corresponding coefficients. Note that both PHIP precursor/product and the solute are present in the sample at all steps of the experiment but the intermolecular cross‐relaxation becomes efficient only when the system obtains net positive magnetization *M_z_* > 0, which happens inside the RF coil.

In PAINTER experiments, the hydrogenation reaction was conducted in the Earth's magnetic field, followed by the sample transfer to the NMR spectrometer and subsequent ^1^H NMR signal acquisition (Figure 1). This experimental approach allowed generation of large quantities of strongly hyperpolarized allyl alcohol. Its hyperpolarization in a low field (LF) does not immediately induce magnetization upon *p*‐H_2_ addition since the initial singlet state of *p*‐H_2_ (|*S*〉) is converted into an analogous singlet state in the PHIP product (|ψ_LF_〉). Subsequent adiabatic transfer to a high field (HF) yields |αβ〉 state (or |βα〉, depending on the spin system parameters [11]), which itself also has net zero magnetization:

(3) $$\begin{array}{c} \;\left|S\right.\rangle =\frac{\left|\alpha \beta \right.\rangle -\left|\beta \alpha \right.\rangle }{\sqrt{2}}\;\overset{\;\mathrm{LF}\;\mathrm{PHIP}\;}{\to }\;\left|\psi_{\mathrm{LF}}\right.\rangle =\left|S\right.\rangle \;\overset{\;\mathrm{adiabatic}\;\mathrm{transfer}\;}{\to }\;\left|\psi_{\mathrm{HF}}\right.\rangle =\left|\alpha \beta \right.\rangle \; \end{array}$$

If strong radiation damping is active, the resulting |αβ〉 state yields RASER and undergoes partial conversion into the |αα〉 state [61], which in turn has non‐zero magnetization and can thus be a polarization donor for the solute via intermolecular NOE. If the solute is a small molecule (as in this work), positive magnetization of the polarization donor results in negative magnetization of the solute. Moreover, as we show below, if this negative polarization on the solute is large enough, it may then trigger RASER of its own. After introduction of the sample into the RF coil and a short waiting time τ_delay_ (<1 s), a train of small flip angle (*θ*) RF pulses with a repetition time *t*_R_ was applied to monitor the evolution of NMR signals (Figure 1). As discussed further in the text, in some experiments, a long waiting time τ_delay_ (>30 s) was implemented to allow unperturbed evolution of PHIP product RASER and PRINOE before the application of a single RF pulse. Detailed experimental procedure may be found in Section S1.

### 2.1 Induction of RASER of a Solute

**FIGURE 2:**
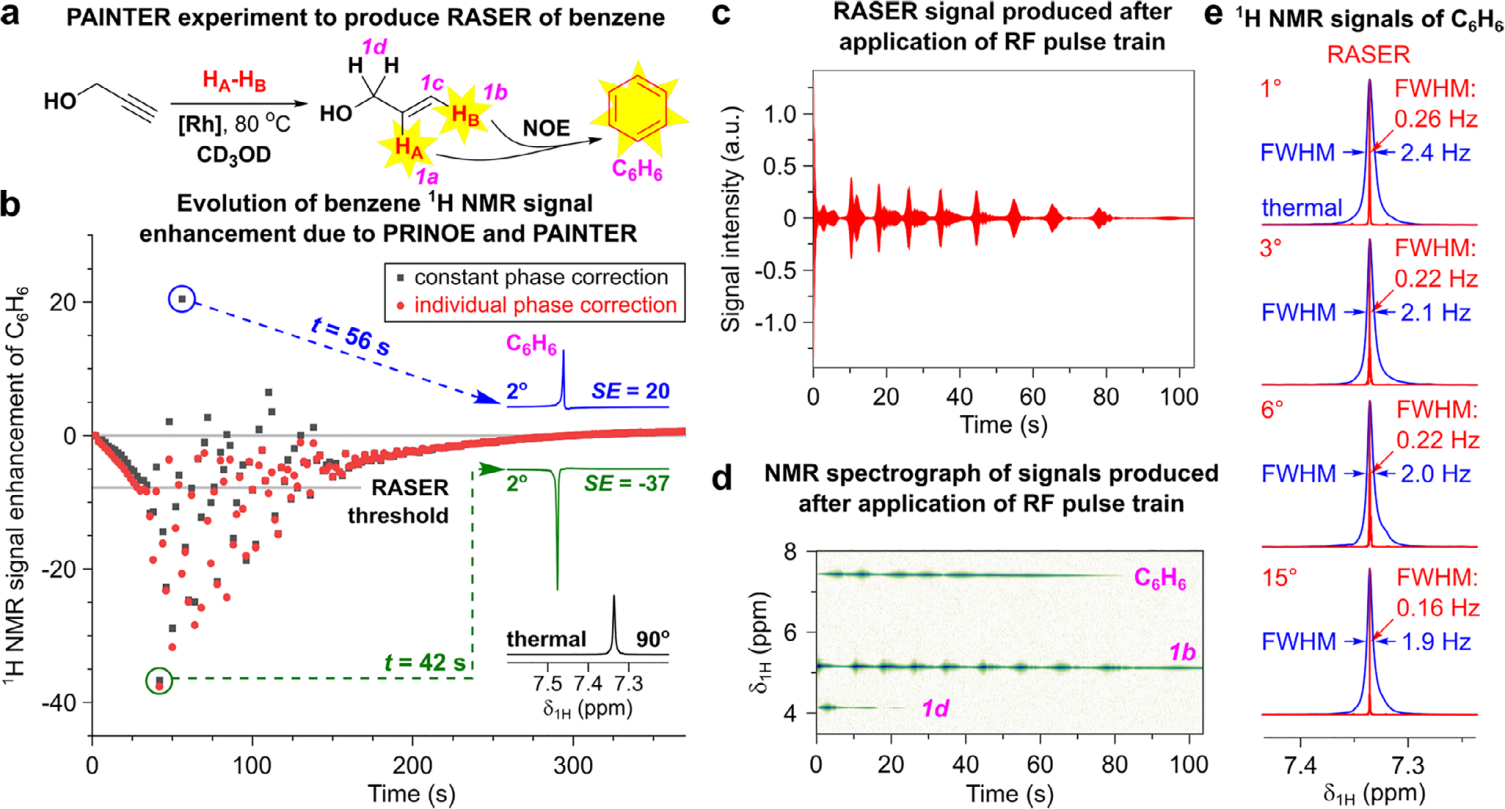
(a) Reaction scheme of pairwise addition of *p*‐H_2_ to **1′** yielding HP **1** with subsequent transfer of polarization to benzene via NOE. (b) Kinetics of PRINOE ^1^H NMR signal enhancement (*SE*) of benzene obtained by integration after applying individual (red) or constant (black) phase correction parameters. For individual correction, the phase of each spectrum in the series was adjusted to make the benzene signal fully emissive, while constant phase correction used the same parameters applied to the whole pseudo‐2D set (using thermal signals of benzene and **1** in the end and ALTADENA signals of **1** in the middle of pseudo‐2D series as references). Insets: regions of ^1^H NMR spectra showing enhanced signals of benzene with constant phase correction (blue, t = 56 s, and green, t = 42 s, 2° pulse) and signal of benzene after relaxation to thermal equilibrium (black, 90° pulse). (c) ^1^H RASER signal acquired after application of a pulse train consisting of 32 RF pulses with a *θ*  *=*  2° and *t*_R_ = 2 s. (d) ^1^H NMR spectrograph produced from the RASER signal presented in panel (c). (e) ^1^H NMR signals of benzene after its RASER was triggered by a single RF pulse with variable flip angle *θ* (red, magnitude mode; frequency drift correction applied) and after thermal equilibrium was reached (blue, real part of the spectra). The corresponding RASER and thermal NMR signals are superimposed so that their maximum intensities match. See Section S1 and Figure S2 for more details on the production of frequency‐drift corrected NMR spectra and spectrographs.

First, PRINOE hyperpolarization of benzene was investigated using **1** as a polarization donor (Figure 2a). Analogous to our previous study [61], series of ^1^H NMR spectra were recorded utilizing 2° RF pulses with a repetition time *t*_R_ of 2 s to monitor NMR signal enhancement (*SE*). The time evolution of the ^1^H NMR signal of benzene was sharply different compared to the previously reported PRINOE studies with ethyl acetate polarization donor [61] (Figure 2b). After an initial smooth PRINOE polarization buildup, we observed strong oscillations in the intensity of benzene NMR signal for almost 2 min, accompanied by phase variation. This period of signal oscillations was followed by smooth polarization decay to thermal equilibrium. We attribute this anomalous behavior to the emergence of RASER on benzene after its PRINOE polarization exceeds the RASER induction threshold. This finding was further supported by an additional experiment, where the RASER NMR signal of benzene persisted for ca. 90 s after the train of 32 RF pulses (with *θ* = 2° and *t*_R_ = 2 s) was applied (Figure 2c,d). To the best of our knowledge, this is the first manifestation where parahydrogen addition and intermolecular NOE polarization transfer engendered RASER (PAINTER).

It was found that application of RF pulses plays a crucial role in the emergence of PAINTER effects. RASER of benzene solute was not observed when no RF pulse was applied, but a single timely positioned 1–15° pulse was sufficient to induce RASER (Table S4). Apparently, whereas the achieved negative magnetization of benzene is above the RASER threshold, *T*_1_ relaxation is not sufficient to trigger RASER (unlike the case of **1** [62]). The resulting benzene RASER signals were significantly narrowed to a full width at half maximum (FWHM) below 1 ppb (0.3 Hz at 7.05 T), while conventional ^1^H NMR signals of benzene in the same samples typically exhibited FWHM of 2–2.5 Hz (Figure 2e). Single pulses with larger flip angles yielded only marginal RASER activity of benzene (Table S4 and Figure S7). Interestingly, the RASER signals of benzene typically exhibited minor satellite artifacts (Figure S8); we attribute them to radiation damping effects, as similar satellites were also observed for RASER‐active protons of **1**, and the distance between the satellites was consistently ca. 10–10.5 Hz.

**FIGURE 3:**
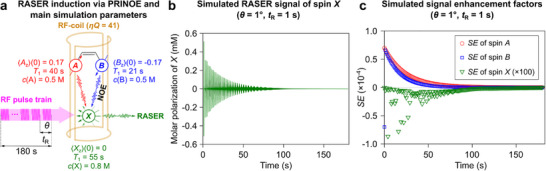
(a) Schematic representation of spin order transfer from ALTADENA‐hyperpolarized spins *A* and *B* to spin *X* via PRINOE and some simulation parameters. (b) Simulated RASER signal of spin *X* induced using 1° flip pulses applied every 1 s. (c) Enhancement factors (*SE*) for simulated NMR signals of *A*, *B*, and *X* (*SE* values for spin *X* are scaled by a factor of 100 for clarity).

Furthermore, we were able to reproduce PAINTER effect via simulations. A simplified 3‐spin system consisting of two spins A and B, representing *p*‐H_2_‐nascent protons, and an isolated spin *X* representing ^1^H nuclei of benzene, was considered (Figure 3a). For solute spin *X*, a concentration of 0.8 M was assumed, while for HP donor spins *A* and *B*, a value of 0.5 M was used since the typical chemical conversion of **1′** into **1** was 64 ± 4% in the experiments. The evolution of magnetization of spins *A*, *B*, and *X* was described by the modified Solomon equations, which account for the possibility of RASER considering coupling of all the spins via cross‐relaxation [62] (see Section S5). Once every *t*_R_, the magnetization vectors were instantaneously flipped by an angle *θ*. A single simulation indeed reveals RASER activity of spin *X* and chaotic behavior of its *SE* (Figure 3b,c).

### 2.2 Impact of RF Pulses on PAINTER Effect

**FIGURE 4:**
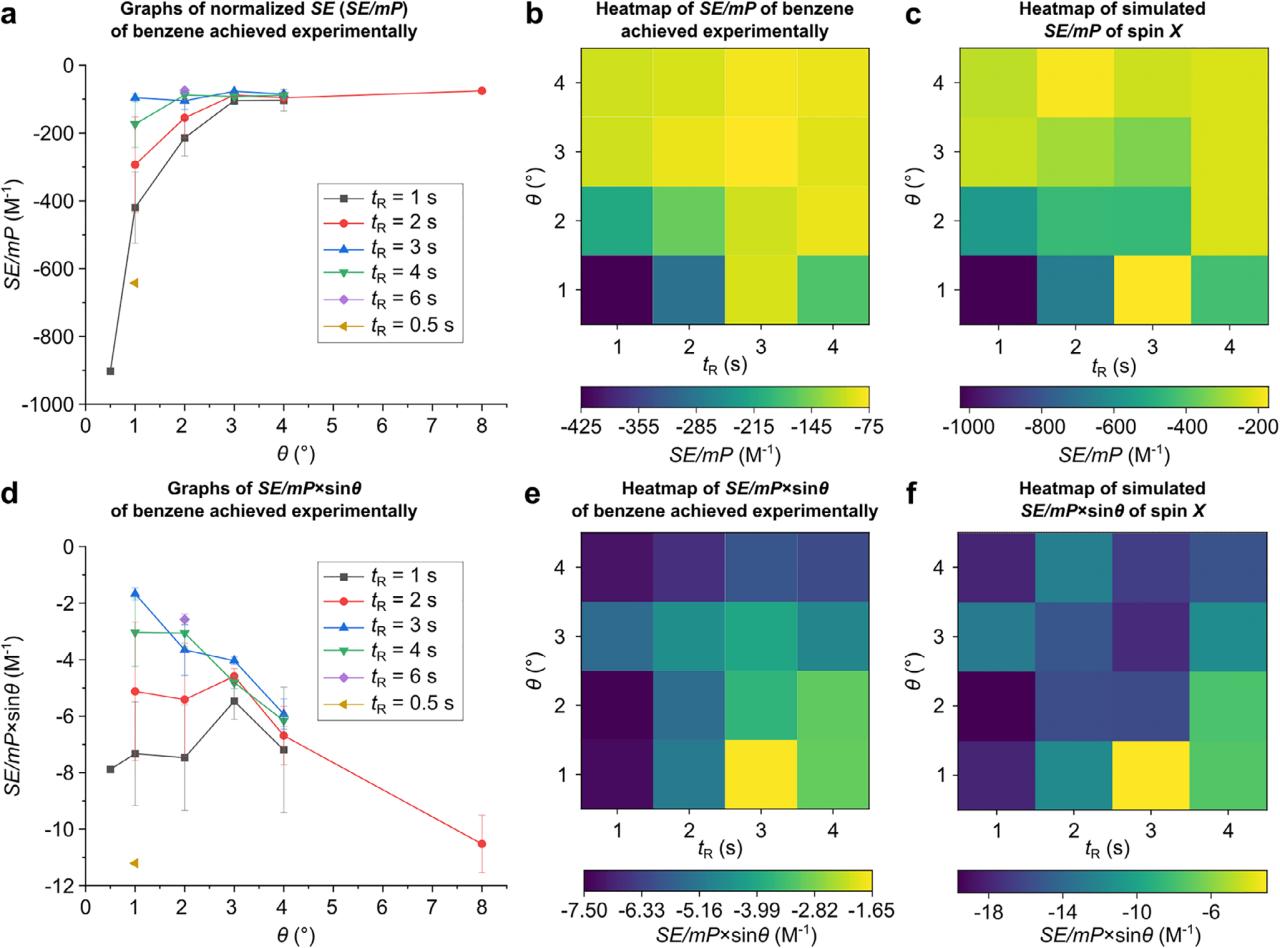
(a) Normalized enhancement factors (*SE*/*mP*) of benzene NMR signals achieved in the PAINTER experiments with varying *θ* and *t*_R_ parameters. (b) Data presented in panel (a) in the format of a heatmap. (c) Heatmap of *SE*/*mP* of spin *X* achieved in the PAINTER simulations with varying *θ* and *t*_R_ parameters. (d) Normalized enhancement factors corrected by the flip angle (*SE*/*mP*·sin*θ*) of benzene NMR signals achieved in the PAINTER experiments with varying *θ* and *t*_R_ parameters. (e) Data presented in panel (d) in the format of a heatmap. (f) Heatmap of *SE*/*mP*·sin*θ* of spin *X* achieved in the PAINTER simulations with varying *θ* and *t*_R_ parameters.

We further investigated the effects of RF pulse trains parameters (in particular, flip angle *θ* and repetition time *t*_R_) on the resultant *SE* of a solute, both experimentally and via simulations. For the experimental study, a series of PAINTER experiments with varying *θ* and *t*_R_ were conducted. In order to mitigate the chaotic changes in the benzene signal intensity (caused by RASER induced on benzene) that may lead to outlier data points, here *SE* values from the 10 most intensive scans in each series were used for analysis. To account for the variations in molar polarization (*mP*) of **1** between different samples (which should proportionally affect benzene polarization achieved through NOE transfer), the *SE* values were divided by initial molar polarizations of **1** in the corresponding samples. The obtained normalized *SE* values (*SE*/*mP*) were averaged for each pair of *θ* and *t*_R_ (the detailed data analysis procedure can be found in Section S3). It was found that *SE*/*mP* of benzene tends to increase with the reduction of both *θ* and *t*_R_ (Figure 4a,b). The same trend was observed in the simulated data (Figure 4c). Comparison of PRINOE kinetic curves measured experimentally at different RF pulse train parameters showed that the initial PRINOE polarization buildup did not depend on *θ* and *t*_R_–*SE* of ca. –8 is consistently achieved after 30–35 s. This indicates that RF pulsing rate affects RASER sustainability rather than NOE efficiency. In turn, the effect of *θ* can be rationalized in a way that if the RF pulses are too strong, they may lead to faster consumption of longitudinal magnetization of benzene.

We note that the simulations predict 2–4 times higher *SE*/*mP* values than observed experimentally. This may be explained by the fact that in experiments the sample is still hot after the hydrogenation, decreasing the NOE polarization transfer efficiency, which favors lower temperatures [63]; additionally, NOE interactions of **1** with the solvent are not taken into account. Furthermore, RASER of spin *X* is induced almost right after the start of the simulations (Figure 3c), unlike in real experiments. This may be attributed to the strong distant dipolar fields (DDF) [64, 65] hindering dipole–dipole interactions between **1** and benzene, postponing the polarization transfer via cross‐relaxation. The DDF arises in the samples immediately after their introduction into the NMR probe as a result of high molar polarization of **1**. Such a difference between the experiments and corresponding simulations has been observed earlier for intramolecular RASER induction via PRINOE [62]. Further studies are warranted to reveal the effect of distant dipolar fields on PAINTER in details.

When a flip angle of 1° and a repetition time of 1 s were used, *SE* of –42 ± 11 (equivalent to *P*_1H_ ≈ 0.1% or molar polarization *mP*(C_6_H_6_) ≈ 0.8 mM) was typically achieved for benzene in the experiments. While this constitutes only under 0.01 of initial molar polarization of **1** (≈101 mM, see Table 1), even this was sufficient to induce RASER of benzene due to its high concentration.

**TABLE 1:** PHIP reaction schemes and corresponding average chemical conversion of **1′–4′** to **1–4** (*X*), initial molar polarization of HP donors **1–4** (*mP*), maximum ^1^H NMR signal enhancement factors of benzene (*SE*), and maximum normalized signal enhancement factors (*SE*/*mP*) obtained in PAINTER experiments employing RF pulse trains with a flip angle of 1° and repetition time of 1 s. Note that *SE* and *SE*/*mP* values were obtained using averaging of 10 maximal data points in each experiment; the detailed calculation procedure can be found in Section S3. Although *X* and *mP* values should not depend on RF pulsing protocol, for **1** here, we used only data from the experiments with 1°/1 s pulse trains for a consistent comparison with other HP donors. Analogous data with *mP* of benzene is also provided in Table S6.

HP donor	PHIP reaction scheme	*X*, %	*mP*(donor), mM	*SE*(C_6_H_6_)	*SE*(C_6_H_6_)/*mP*, M^−1^
**1**	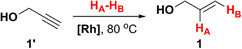	64 ± 4	101 ± 1	−42 ± 11	−420 ± 105
**2**	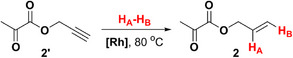	63 ± 1	75 ± 3	−6 ± 1	−82 ± 13
**3**	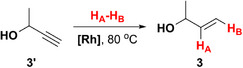	44 ± 2	76 ± 8	−6.3 ± 0.8	−84 ± 16
**4**	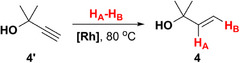	60 ± 11	107 ± 13	−14 ± 5	−125 ± 41

Additionally, the signal enhancement corrected by a flip angle *SE*/*mP*·sin*θ* was analyzed (Figure 4d,e); this value is representative of the transverse magnetization value $\left\langle {\hat{X}}_{tr}\right\rangle$. For pulsing protocols with longer *t*_R_ delays (3 and 4 s), the *SE*/*mP*·sin*θ* depends mainly on the value of *θ* and is roughly the same for different *t*_R_. Similarly, at *θ* = 3 and 4°, the *SE*/*mP*·sin*θ* value does not show significant dependence on *t*_R_. However, when small flip angles *θ* = 1° and 2° are applied, the achieved *SE*/*mP*·sin*θ* increases if the repetition time *t*_R_ is shortened. These trends are supported by simulations (Figure 4f; for more details regarding the simulations, see Section S5). Further studies are warranted to completely understand the observed effects of RF pulses on the RASER of solute.

### 2.3 HP Donor Effect on PAINTER Efficiency

Next, we investigated a number of HP donors structurally similar to allyl alcohol (namely, allyl pyruvate (**2**), 3‐buten‐2‐ol (**3**), and 2‐methyl‐3‐buten‐2‐ol (**4**), see Table 1) in PAINTER experiments using benzene solute. As discussed above, the lower *θ* and *t*_R_ are, the larger enhancement of the benzene NMR signals (i.e., stronger benzene RASER) is possible to achieve. However, we chose to employ RF pulse trains with a flip angle of 1° and a repetition time of 1 s as a compromise solution between temporal and spectral resolutions, as reduction of *t*_R_ is achieved by decreasing the number of sampling points in the FID signal. Oscillations of the NMR signal of benzene were observed in the case of all HP products **1–4** used as polarization donors (Figure S9), proving that RASER was induced on the solute. However, **1** provided the overall strongest NMR signal enhancement, in terms of both absolute (*SE*) and normalized (*SE*/*mP*) values (Table 1). When NMR signals were acquired continuously for 104 s after application of 1°/1 s RF pulse trains until the maximum *SE* of benzene was reached, only **1** provided strong, long‐lasting (over 80 s) and reproducible RASER of benzene (Table S5). In the cases of **3** and **4**, RASER observation was inconsistent. Moreover, in the case of **3**, only very weak and short‐lasting RASER could be detected. Thus, from the four allylic compounds tested in this study, **1** proved to be the most efficient polarization donor in PRINOE and PAINTER experiments. This result correlates with the fact that for **2** and **3**, lower HP donor molar polarizations were obtained compared to those for **1** and **4** (Table 1). Moreover, *1a* proton has longer *T*_1_ relaxation time than *2a*, *3a*, and *4a* (see Table S3), providing more efficient PRINOE polarization buildup (note that these protons have the strongest positive polarization and thus contribute to PRINOE more than other groups of protons). Lower efficiency of intermolecular PRINOE transfer from **3** and **4** to benzene may also be attributed to the fact that these compounds contain additional methyl groups *3e* and *4d* that act as polarization sinks through intramolecular NOE (Figure S10) [62]. This is further supported by the fact that **4** has greater molar polarization than **1** (Table 1) but yields weaker RASER of benzene (Table S5). Finally, the smaller size of **1** may provide more efficient intermolecular dipolar polarization transfer via closer contact between **1** and benzene; this factor likely also contributes to low efficiency of **2** as pyruvoyl moiety prevents efficient contact between benzene and HP allyl moiety of **2**.

Apart from the solute and the protons in the HP donors, there are also residual protons in the solvent (CHD_2_OD in methanol‐d_4_) and labile OH protons, which receive polarization from the HP donors via cross‐relaxation, presenting additional pathways competing with NOE transfer to the solute. The maximal *SE* for the CHD_2_ protons was ca. –10, demonstrating greater enhancement than observed earlier in PRINOE experiments using HP ethyl acetate as an HP donor [61]. Similar enhancements were observed for the OH protons when **1**, **3,** or **4** HP donors were used, whereas in the case of **2** no signal enhancement for the labile protons was observed. This indicates that polarization of OH protons is likely induced via intramolecular NOE, while they are bound to the HP donor molecules **1**, **3,** or **4**, which have a hydroxyl group.

### 2.4 Range of Targets for PAINTER Effect

Furthermore, experiments with several other solutes, beyond benzene, were conducted using **1** as a polarization donor to broaden the scope of the observed PAINTER effects. The results obtained using 1°/1 s RF pulse train protocol are summed up in Table 2. PRINOE effects were obtained for ethyl pyruvate (EtPyr), furan, cyclohexane (CyH), and hexamethyldisiloxane (HMDSO). For EtPyr and furan no oscillations in the PRINOE kinetics were observed (Figure S11), indicating that RASER on these solutes is not induced, as a result of lower proton payload contributing to an NMR signal of interest (3 and 2, respectively, vs. 6 for benzene) and (for EtPyr) lower molar polarization of **1**. CyH and HMDSO have a greater number of equivalent protons, *N*, than benzene (12 and 18, respectively). As the critical *SE* values (*SE*_crit_), above which RASER is initiated, are inversely proportional to *N*, the experimentally observed *SE*_crit_ ≈ –8 for benzene (*N* = 6) gives rough estimates of *SE*_crit_ ≈ –4 for CyH (*N* = 12) and *SE*_crit_ ≈ –2 for HMDSO (*N* = 18). As a result, HMDSO exhibited oscillations in the intensity of its ^1^H NMR signal in some experiments (Figure S10), despite having a lower average *SE* = –6 ± 2 than furan; however, these oscillations were short and of low intensity. Moreover, reproducibility of PAINTER for HMDSO was quite poor (2 out of 5 tests yielded RASER of the solute), indicating that the maximum achieved PRINOE polarization for HMDSO is only slightly above the RASER threshold. Oscillations of the ^1^H NMR signal (i.e., RASER) of cyclohexane were reproducible but weaker than those of benzene (Figure S11), arguably because of the shorter *T*_1_ of the corresponding ^1^H nuclei.

**TABLE 2:** Average chemical conversion of **1′** to **1** (*X*), initial molar polarization of HP donor **1** (*mP*), maximum ^1^H NMR signal enhancement factors of solutes (*SE*), maximum normalized signal enhancements (*SE*/*mP*), and *T*_1_ relaxation times for different solutes. *T*_1_ values were measured at 7.05 T using an inversion‐recovery protocol under H_2_ pressure after hydrogenation. Analogous data with *mP* of the solutes is also provided in Table S7.

Solute	*X*, %	*mP*(1), mM	*SE*(solute)	*SE*(solute)*/mP*(1), M^−1^	*T*_1_, s
Benzene	64 ± 4	101 ± 1	−42 ± 11	−420 ± 105	56 ± 1
Furan^a^	60 ± 3	116 ± 9	−13.8 ± 0.8	−120 ± 14	92 ± 6 [61]
EtPyr^b^	31 ± 3	66 ± 6	−3.5 ± 0.6	−53 ± 6	−
CyH	56 ± 10	108 ± 6	−27 ± 28	−242 ± 240	18.4 ± 0.6
HMDSO^c^	62 ± 15	104 ± 14	−6 ± 2	−60 ± 21	10.0 ± 0.4
HMDSO^d^	59 ± 8	87 ± 3	−1.87 ± 0.09	−21.5 ± 0.8	

^a^ Data for protons in the α‐position.

^b^ Data for the methyl group of the pyruvate fragment.

^c^ Data from the spectra acquired in the experiments with ^1^H RASER induced on HMDSO.

^d^ Data from the spectra acquired in the experiments without ^1^H RASER induced on HMDSO.

**FIGURE 5:**
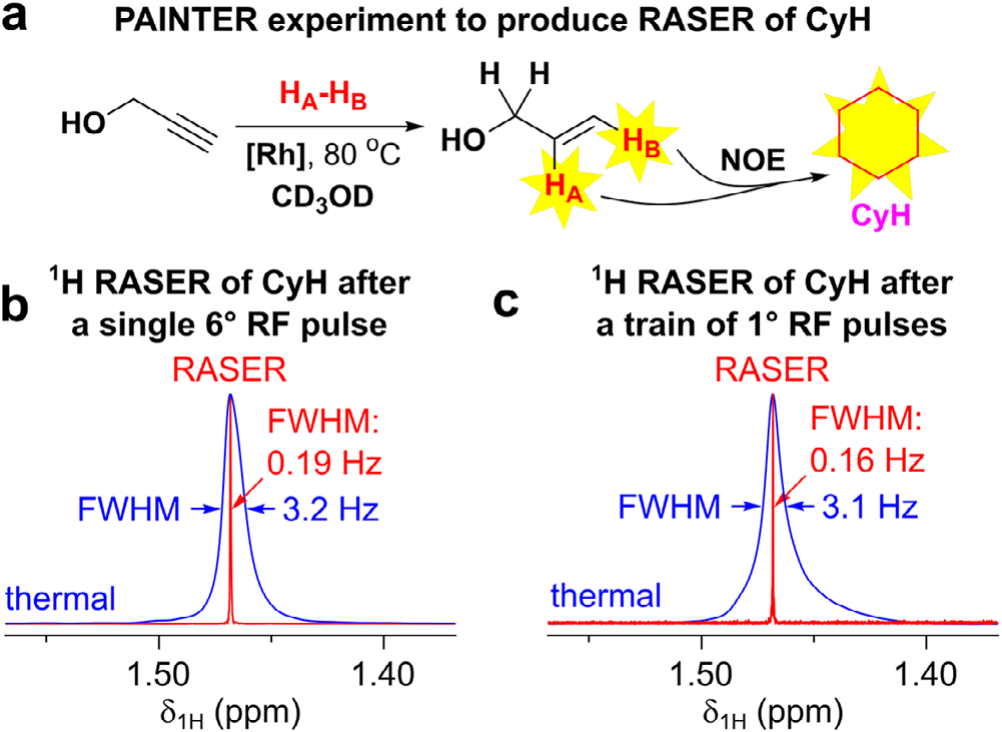
(a) Reaction scheme of pairwise addition of *p*‐H_2_ to **1′** yielding HP **1** with subsequent transfer of polarization to cyclohexane (CyH) via NOE. (b) ^1^H NMR signal of CyH after its RASER was triggered by a single 6° RF pulse (red, magnitude mode; frequency drift correction applied) and after thermal equilibrium was reached (blue, real part of the spectrum). (c) ^1^H NMR signal of CyH after its RASER was triggered by a train of 1° RF pulses applied with a 1 s interval (red, magnitude mode; frequency drift correction applied) and after thermal equilibrium was reached (blue, real part of the spectrum). In panels (b) and (c), the corresponding RASER and thermal NMR signals are superimposed so that their intensities match.

The possibility of direct RASER detection using a 104 s‐long NMR signal acquisition applied after either a single 6° RF pulse or a 1°/1 s RF pulse train was explored for cyclohexane and HMDSO. Only the former solute demonstrated RASER effects with FWHM of RASER‐derived ^1^H NMR signals below 0.2 Hz (Figure 5). The RASER activity was not particularly strong and lasted ∼20 s (Tables S8 and S9). An attempt to achieve RASER of CyH without applying RF pulses was unsuccessful.

### 2.5 PRINOE on Benchtop NMR

Finally, the possibility of RASER induction on the solutes was investigated using a benchtop 1.4 T NMR spectrometer rather than the 7.05 T instrument used in all experimental studies described above. The most efficient HP system, featuring HP donor **1** and benzene solute, was chosen, and the PRINOE kinetics were probed using 1° RF pulses applied every 1.07–1.08 s. Mean signal enhancement values *SE* = –46 ± 2 and *SE*/*mP* = –820 ± 360 M^−1^ were obtained for benzene (note that as thermal polarization of spins is proportional to magnetic field, *SE* at 1.4 T amounts to *P*_1H_ of only ≈0.02% vs. ≈0.1% achieved at 7.05 T, even though the *SE* factors themselves are similar). No pronounced oscillations of PRINOE intensity were detected (Figure S12), indicating that the achieved magnetization of benzene is not high enough to induce RASER in these conditions. This result can be, at least in part, attributed to the ca. 4.4‐fold difference in *Q* factors of the ^1^H probes of these two NMR spectrometers (see Section S4). Additionally, the chemical conversion of **1′** to **1** was almost 100% in these experiments (see Section S10), which may influence the characteristics of RASER of **1** and, subsequently, polarization dynamics of benzene. We note, however, that careful optimization of experimental parameters may enable PAINTER effects with benchtop NMR spectrometers in the future.

## 3 Conclusion

The feasibility of parahydrogen addition and intermolecular NOE transfer engendered RASER (PAINTER) effect of the solute molecules was demonstrated. This was achieved by utilizing strongly hyperpolarized RASER‐active allylic compounds as donors of polarization, namely allyl alcohol (**1**), allyl pyruvate (**2**), 3‐buten‐2‐ol (**3**), and 2‐methyl‐3‐buten‐2‐ol (**4**). The presented approach enables RASER induction on inert solute molecules that do not chemically interact with either *p*‐H_2_ or polarization donors **1–4**. The parameters of the RF pulsing protocol had a pronounced effect on the induced RASER intensity, with more frequent pulsing at smaller flip angles yielding stronger RASER for the solutes. These findings were also supported by simulations. The RASER‐active solutes featured enhancement of their NMR signals and the linewidths below 0.3 Hz, making them highly resolved and intense, considering also that the signals of thermally polarized species are almost invisible, since only tiny RF pulses are applied before acquisition of RASER. It is worth noting that while PAINTER effect discussed in this work involves intermolecular transfer, it may also encompass previously described [62] intramolecular RASER induction (in this case, with “I” standing for “intramolecular”).

While SABRE‐Relay [66] and PHIP‐X [42] extended the scope of molecules hyperpolarized via parahydrogen‐based techniques to those that participate in proton exchange, PRINOE further expands the range of possible targets, making chemical exchange unnecessary and allowing, in theory, hyperpolarization of any molecule. Here, we already achieved a sufficiently high signal enhancement to even induce RASER on such solutes that leads to 10–20 times smaller width of their NMR signals. While in this work the scope of RASER‐active solutes is limited to molecules with a high payload of chemically equivalent protons (benzene and cyclohexane) in high concentrations of 0.8 M, we believe that it would be possible to expand it utilizing RF coils with higher *Q* factors, for example, the recently reported parametrically pumped wireless detectors [67]. Employing such RF coils may also render pulsing unnecessary for generating RASER signals from solutes. Another potential prospect is the induction of RASER of heteronuclei via PRINOE; this would allow the detection of solutes without interference from RASER‐active polarization donors. Furthermore, the approach presented here, in principle, is not limited to parahydrogen‐based hyperpolarization techniques—it should be possible to induce RASER via intermolecular SPINOE transfer from species hyperpolarized via dissolution DNP or SEOP [43, 45].

## Conflicts of Interest

EYC discloses a stake of ownership in XeUS Technologies LTD and PerXeus Technologies Inc.

## Supporting information




**Supporting File 1**: The authors have cited additional references within the Supporting Information[68, 69, 70, 71, 72, 73, 74, 75].

## Data Availability

The data that support the findings of this study are available from the corresponding author upon reasonable request.
