# Supplementary material for: Intermolecular Nuclear Spin Hyperpolarization Transfer via Cross‐Relaxation Triggers RASER of Solute Molecules
Source: Angew Chem Int Ed Engl. 2026 Mar 26;65(19):e2865398. doi: 10.1002/anie.2865398 (PMC13134604; doi:10.1002/anie.2865398)
Supplement: Supplementary file 1 — Supporting File 1: The authors have cited additional references within the Supporting Information[68, 69, 70, 71, 72, 73, 74, 75]. [file ANIE-65-e2865398-s001.pdf]

Supporting Information for

Intermolecular Nuclear Spin Hyperpolarization Transfer via Cross-Relaxation Triggers RASER of Solute Molecules

Ivan A. Trofimov,*,# Anna P. Yi,# Oleg G. Salnikov,* Andrey N. Pravdivtsev, Henri de Maissin, Eduard Y. Chekmenev, Jan-Bernd Hövener, Andreas B. Schmidt, and Igor V. Koptyug

Table of contents

Section S1. Experimental and data processing details 2
Section S2. NMR parameters of precursors 1'–4' and hydrogenation products 1–4 4
Section S3. Calculation of conversions, signal enhancements and polarizations in PRINOE experiments 5
Section S4. Radiation damping measurements, calculation of ηQ product and threshold magnetization 6
Section S5. Simulations of RASER induction via PRINOE 7
Section S6. ^{1}H RASER of benzene triggered by a single RF pulse 10
Section S7. ^{1}H RASER of benzene triggered by RF pulse trains 14
Section S8. Comparative efficiency of hyperpolarized donors 1–4 for PRINOE and RASER induction 16
Section S9. PRINOE studies of other solutes beyond benzene 18
Section S10. PRINOE experiments at 1.4 T benchtop NMR spectrometer 21
References 22

Section S1. Experimental and data processing details

Sample preparation

Commercially available unsaturated substrates (propargyl alcohol (Sigma-Aldrich, 99%), 3-butyn-2-ol (Alfa Aesar, 98%), 2-methyl-3-butyn-2-ol (Sigma-Aldrich, 98%)), benzene (Chemical Line, ≥99%), cyclohexane (Sigma-Aldrich, ≥99%), furan (Sigma-Aldrich, ≥99%), ethyl pyruvate (Acros Organics, 98%), methyl (*S*)-lactate (Acros Organics, 97%), acetonitrile (Concord Technology, HPLC grade), pyruvic acid, urotropine, glycine, choline chloride (Thermo Scientific, 99%), bis(norbornadiene)rhodium(I) tetrafluoroborate ($[Rh(nbd)_2]BF_4$, nbd = norbornadiene, Umicore, 99.9%), 1,4-bis(diphenylphosphino)butane (dppb, Sigma-Aldrich, 98%), methanol-d_4 (Zeotope, 99.8% D) and ultrapure hydrogen (>99.999%) were used as received. Propargyl pyruvate was synthesized according to the previously published procedure.[1] Hexamethyldisiloxane was kindly provided by Georgy E. Salnikov (Vorozhtsov Novosibirsk Institute of Organic Chemistry SB RAS).

Hydrogen gas was enriched with p-H_2 using a parahydrogen generator based on a closed-cycle helium cryostat (CryoPribor, CFA-200-H2CELL) and a cryocompressor (Sumitomo, Zephyr HC-4 A). Ortho-para conversion was conducted over hydrated iron oxide FeO(OH) (Sigma-Aldrich, 371254) catalyst. The p-H_2 enrichment of 94% was achieved according to gas-phase NMR quantification.

At first, the mixture of $[Rh(nbd)_2]BF_4$ and dppb was dissolved in methanol-d_4. The amounts of reactants were calculated to get the concentration of Rh complex of 10 mM; dppb was taken in ~2% molar excess with respect to $[Rh(nbd)_2]BF_4$. The solution was left for ~30 min with periodic mixing to ensure formation of $[Rh(nbd)(dppb)]BF_4$ complex. The resultant solution (0.5 mL aliquots) was placed in standard 5 mm Wilmad NMR tubes tightly connected with ¼ in. outer diameter PTFE tubes. Before conducting an experiment, a portion of one of the unsaturated precursors **1'**–**4'** was added to an NMR tube as well as a portion of a desired solute; the amount of both additives was calculated so that its concentration in the resultant solution was 0.8 M (neglecting the slight dilution due to own volume of the added substances). The solution was thoroughly mixed afterwards to ensure homogeneous distribution of the dissolved compounds.

ALTADENA PRINOE protocol

The experiments were performed in 5 mm standard NMR tubes at 7.05 T Bruker AV 300 NMR spectrometer equipped with a 5 mm PH DUL 300S1 C-H-D-05 NMR probe. The Q factor measurements for the probe are discussed in Section S4. The scheme of the experimental setup is presented in Figure S1. The gas lines were purged with p-H_2 for ~5 min. The sample was connected to the gas lines and pressurized to 6.2 bar (regulated by a 75 psig safety valve) while the bypass valve was opened. The sample was heated to 80 °C in a beaker with hot water for 30 s. Then, p-H_2 bubbling was initiated by closing the bypass valve; the gas was bubbled at a 100 standard cubic centimeters per minute (sccm) gas flow rate for 15 s. The gas flow rate was regulated using a mass flow controller (Brooks Instrument, model 5850E). Next, the sample was rapidly taken out from the beaker, dried with a paper towel, and placed into the NMR probe of the NMR spectrometer. The temperature of the NMR probe was set at 297 K. NMR signal acquisition was started right after the end of p-H_2 bubbling (7–8 s before the sample was placed into the NMR probe). A series of NMR spectra were then acquired using a small flip angle (θ, 0.5–8°) excitation RF pulses with short repetition times (t_R, 0.5–4 s).

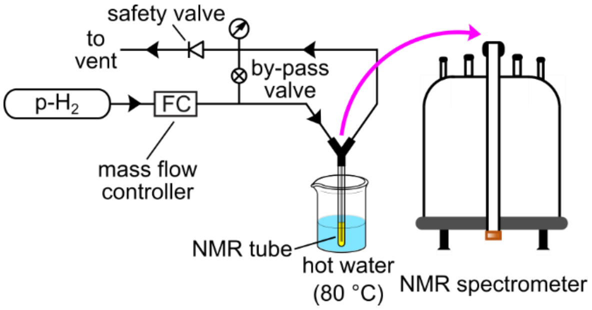

Figure S1. Scheme of the experimental setup used for PRINOE experiments.

The following modifications and specific conditions were implemented in different ALTADENA PRINOE experiments:

1. For acquisition of NMR spectra at 1.4 T, a benchtop SpinSolve Nitrogen 60 NMR spectrometer (Magritek, New Zealand) (Q factor of the ^{1}H channel ~ 165) was utilized.
2. In experiments with direct RASER signal acquisition after application of RF pulse trains, the protocol was almost the same with the exception that the number of 1D spectra in a pseudo-2D set was normalized to stop the train approximately at the moment when the maximal PRINOE enhancement for a particular solute is achieved. The parameters of the pulse train were θ = 1° and t_R = 1 s. After the end of pulse train NMR signal was acquired for 104 s without application of any additional RF pulses.
3. For experiments with a RASER induction using a single RF pulse, a hyperpolarized sample was placed into the NMR probe and stayed there for ca. 64 s. The duration of this delay was chosen so that the maximum of PRINOE enhancement for benzene is achieved. Next, an RF pulse with variable θ was applied to trigger RASER on benzene and an NMR signal was acquired for 0.9 s. This was followed by acquisition of another NMR spectrum for 104 s without the application of an RF pulse (detection of RASER). Although these two spectra were recorded consecutively via queuing in TopSpin spooler, they were in fact separated by an additional delay of 6–7 s.

Production of spectrographs of ^{1}H NMR signals

To obtain time-resolved spectra from the raw RASER data, the following steps were performed. A sliding window was employed to select segments of the time signal. The width of the time window determines the spectral resolution; a longer window yields narrower spectral signals. However, as the duration of the window increases, the time specificity of the window decreases, causing a blurring of the final spectrograph. We found 200 ms to be the best tradeoff. The windowed signal underwent an apodization filter (Hann window) and was zero-filled to 512 points. The signal was then Fourier transformed to obtain the spectrum corresponding to the center of the 200 ms window. This procedure was repeated for each sliding window and a 2D spectrograph image was obtained. The image is displayed in log scale to emphasize the signals with low intensity which are common in RASER spectra besides strong signals. The apparent linewidth of the RASER signals appears broad due to the logarithmic scale employed. Color scaling was normalized to eliminate noise and effectively represent the actual signals.

Note the following features for the presented spectrographs:

- the spectrographs represent magnitude, not specific phase of the signals;

- amplitude color scheme and arbitrary units (a.u.) are consistent in all presented spectrographs.

Frequency drift correction of RASER-derived ^{1}H NMR signals

During RASER, the distant dipolar fields (DDF) produced by a strongly polarized sample weaken, leading to an apparent frequency drift in the resulting NMR spectra. If no field-drift correction is applied, this continuous frequency shift throughout the RASER duration results in very broad peaks in the Fourier-transformed spectrum. To fully exploit the long acquisition time of the RASER signal, a field-drift correction must therefore be applied.

The spectrogram of the RASER signal enables measurement of the amplitude, full width at half maximum (FWHM), and peak position (frequency) of each RASER-active resonance. The peak positions are shown in red in Figure S2. Data points shown in lighter colors correspond to non-RASER signals and were excluded from the fit. We observed that the dominant contribution is an exponentially decaying frequency drift, which we attribute to the DDF. Additional oscillations appear during intense RASER bursts, but these were not included in the model.

Fitting the peak-position curve (in the Fourier domain) allowed us to estimate both the amplitude of the DDF and its relaxation rate. The correction was then applied to the time-domain RASER signal by adjusting the phase according to the integral of the fitted function. The corrected spectrogram validates the efficacy of this procedure, as illustrated in Figure S2: the corrected peak positions (blue) remain stable, and Fourier transformation of the entire field-drift–corrected RASER signal produces the narrow spectral lines shown. To improve the estimation of the FWHM of the field-drift–corrected spectra, a Gaussian apodization window was applied to suppress rapid signal oscillations.

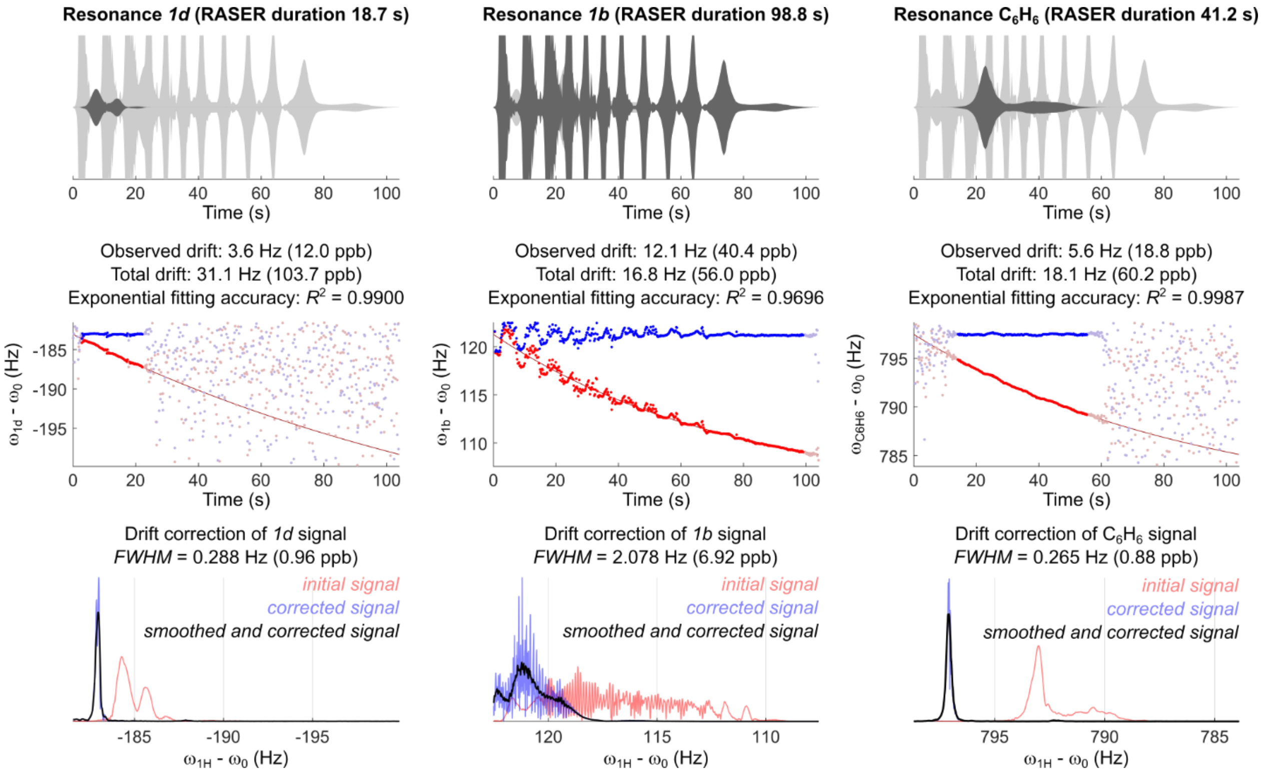

Figure S2. Process of frequency drift correction illustrated on the example of RASER signal acquisition after application of a single 1° RF pulse. Top row: filtered RASER signals corresponding to RASER activity of individual groups of spins (*1d*, *1b* and C_6H_6). Middle row: resonance position graphs fitted by exponential functions (red) and the resulting values after applying frequency drift correction (blue). Bottom row: NMR spectra without drift correction (red), with applied drift correction (blue), and smoothed corrected signals used to determine full width at half magnitude (black). The frequency values are given relative to the central frequency in the NMR spectrum (o1 parameter in TopSpin).

Section S2. NMR parameters of precursors 1'–4' and hydrogenation products 1–4

Table S1. ^{1}H NMR chemical shifts of hydrogenation products **1**–**4** and triple-bond precursors **1'**–**4'**.

Product	Signal	Chemical shift, ppm	Precursor	Signal	Chemical shift, ppm
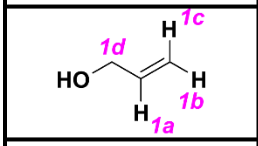	*1a*	5.98	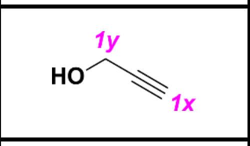	*1x*	2.74
	1b	5.11			
	1c	5.27		*1y*	4.16
	1d	4.07			
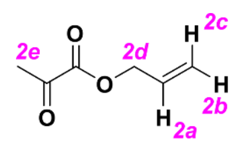	*2a*	5.98	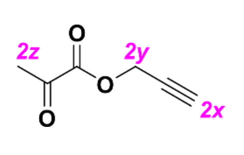	*2x*	3.02, 2.96[a]
	2b	5.26			
	2c	5.37		*2y*	4.85, 4.79[a]
	2d	4.74, 4.68[a]			
	2e	2.43, 1.50[a]		*2z*	2.43, 1.50[a]
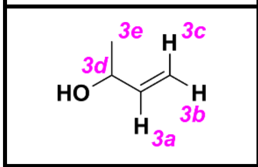	*3a*	5.88	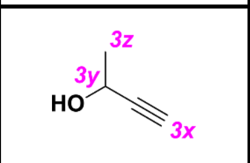	*3x*	2.75
	3b	5.01			
	3c	5.18		*3y*	4.43
	3d	4.22			
	3e	1.22		*3z*	1.39
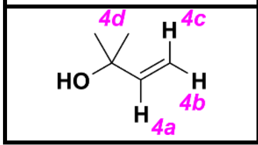	*4a*	5.97	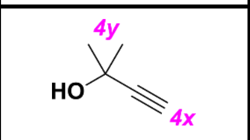	*4x*	2.71
	4b	4.95			
	4c	5.18		*4y*	1.46
	4d	1.26			

[a] In methanol-d_4 compounds **2** and **2'** partially transform into corresponding hemiacetals resulting in additional signals.

Table S2. *J*-coupling constants in the hydrogenation products **1**–**4**.

Product	*J*-constant	J, Hz	Product	*J*-constant	J, Hz
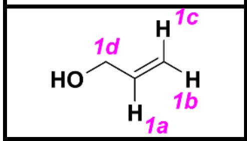	J_{1ab}	10.4	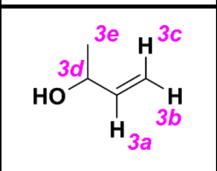	J_{3ab}	10.45
	J_{1ac}	17.15		J_{3ac}	17.15
	J_{1bc}	1.8		J_{3bc}	1.5
	J_{1ad}	5.1		J_{3ad}	5.6
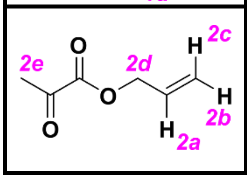	J_{2ab}	10.4		J_{3de}	6.4
	J_{2ac}	17.1	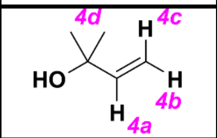	J_{4ab}	10.8
	J_{2bc}	1.8[a]		J_{4ac}	17.4
	J_{2ad}	5.1		J_{4bc}	1.5

[a] The J_{2bc} splitting was not observed in the acquired ^{1}H NMR spectra and was proposed equal to J_{2bc} in **1** because other *J*-coupling constants in the spin system of **2** were found to be identical in these two compounds.

Table S3. Longitudinal relaxation times T_1 of the ^{1}H nuclei in the hydrogenation products **1**–**4** in corresponding solutions after the RASER experiments obtained by inversion recovery technique.

Product	Proton	T_1, s	Product	Proton	T_1, s
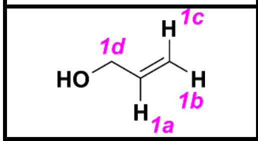	*1a*	40 ± 2	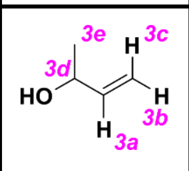	*3a*	25 ± 2
	1b	20.9 ± 0.8		*3b*	14.3 ± 0.4
	1c	20 ± 2		*3c*	13.0 ± 0.5
	1d	10.3 ± 0.4		*3d*	18 ± 2
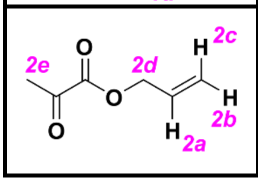	*2a*	27 ± 3		*3e*	4.7 ± 0.1
	2b	13.4 ± 0.4	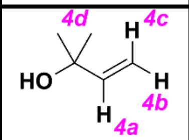	*4a*	26 ± 2
	2c	12.2 ± 0.3		*4b*	14.2 ± 0.3
	2d	5.4 ± 0.2		*4c*	20 ± 2
	2e	–[a]		*4d*	3.81 ± 0.02

[a] Not measured.

Section S3. Calculation of conversions, signal enhancements and polarizations in PRINOE experiments

Conversions X of precursors **1'–4'** to hydrogenation products **1–4** were calculated from the thermal spectra acquired after relaxation of hyperpolarization as $X = \frac{I^P}{I^R + I^P}$, where I^R is the averaged signal of a single proton in the reactant (**1'–4'**) and I^P is the averaged signal of a single proton in the product (**1–4**). Terminal alkyne protons *1x–4x* were typically not considered in calculations of I^R, because their corresponding integrals were typically lower than expected compared to other signals of **1'–4'** (likely due to longer T_1).

In PRINOE experiments, to adjust for varying conversion of precursors **1'–4'** and polarization of donors **1–4** across different experiments, NMR signal enhancement factors normalized to molar polarization of the HP donor (SE_{mP}) were calculated in addition to normal SE. For this, the following steps were undertaken:

1. ^{1}H NMR spectra of thermally polarized samples with precursor **1'** acquired with a 90° RF pulse before hydrogenation were used to calibrate the relationship between the observed NMR integrals I and corresponding molar polarizations $mP = P{\cdot}C$, $k_{mP} = \frac{P \cdot C}{I}$. The integrals of CH_2 group of **1'** were averaged for all samples to obtain $I_{CH2,av}$ and k_{mP} ratio was calculated as $k_{mP} = \frac{2P^{th}[\mathbf{1'}]}{I_{CH_2,av}}$ where [**1'**] = 0.8 M is the nominal concentration of **1'**, $P^{th} = 2.42{\cdot}10^{-5}$ is the thermal polarization of ^{1}H nuclei at 7.05 T and 24 °C.
2. In each pseudo-2D set, batch phase correction was applied using hydrogenation product (**1–4**) ALTADENA and thermal signals as phase references. In each 1D spectrum the full integral of the spectrum was taken as a "total PHIP signal" (I_{PHIP}). This value takes into account that the negative ALTADENA signals are suppressed by RASER, while the positive ones are not; the resultant I_{PHIP} represents the total magnetization in the sample which can be transferred to a solute via NOE. Next, the I_{PHIP} decay curve was fitted using monoexponential decay function $y = A \cdot \exp(-x/T)$. The resulting A value corresponds to the total PHIP signal extrapolated to t = 0 s. The A values from the fits were used to calculate observed molar polarizations (mP) of **1–4** as $mP = \frac{A \cdot k_{mP}}{\sin\theta}$ ($\sin\theta$ factor was introduced to take into account that the HP spectra were acquired with varied low-angle pulses θ, while the thermal spectra were acquired with a 90° pulse).
3. Next, for each 1D spectrum in a pseudo-2D set, where PRINOE hyperpolarization of a solute was observed, the individual phase correction parameters were applied to make solute signal fully emissive (or close to this, if the signal lineshape is somewhat distorted). Signal enhancements (SE) for the solute were calculated by comparing its HP signal intensity I^{HP} with its thermally polarized signal intensity I^{th} measured after the relaxation of hyperpolarization with a 90° RF pulse ($SE = \frac{I^{HP}}{I^{th} \cdot \sin\theta}$). In each pseudo-2D set, 10 data points with the highest SE were taken and used for the following assessments. 10 data points were used with the goal of correcting for the pronounced chaoticity of evolution of RASER intensity induced on the solute.
4. Next, these 10 SE values were divided by an mP value of a hyperpolarized donor **1–4** for a particular experiment to obtain the normalized signal enhancement (SE_{mP}) values, $SE_{mP} = \frac{SE}{mP}$. Molar polarization mP was thus used as a measure of effective ALTADENA signal available for NOE transfer to the solute. Since (*i*) the solute SE is expected to be proportional to the effective ALTADENA signal and (*ii*) there are some variations in molar polarization between the different samples, this procedure helps to eliminate this unwanted randomizing factor and thus decrease the standard deviation of data.
5. Next, the resulting SE_{mP} values were taken for several experiments performed using the same pulsing protocol (θ, t_R) and averaged. Altogether, n experiments provided ($10{\cdot}n$) SE_{mP} values which were used to estimate the mean SE_{mP} and the standard deviation. The n values for experiments with variation of RF pulsing parameters are provided in the table below:

**8°**			*n* = 2			
**4°**		*n* = 2	*n* = 2	*n* = 2	*n* = 2	
**3°**		*n* = 2	*n* = 2	*n* = 2	*n* = 2	
**2°**		*n* = 3	*n* = 9	*n* = 3	*n* = 2	*n* = 2
**1°**	*n* = 1	*n* = 2	*n* = 5	*n* = 3	*n* = 3	
**0.5°**		*n* = 1				
**θ / t_R**	**0.5 s**	**1 s**	**2 s**	**3 s**	**4 s**	**6 s**

The n values for experiments with HP donors **2–4**:

**HP donor**	**2**	**3**	**4**
n	*n* = 2	*n* = 2	*n* = 3

The n values for experiments with various solutes:

**Solute**	**Furan**	**EtPyr**	**CyH**	**HMDSO (RASER)**	**HMDSO (no RASER)**
n	*n* = 2	*n* = 2	*n* = 3	*n* = 2	*n* = 3

When the effect of the parameters of RF pulsing (θ and t_R) on the induced RASER is considered, from the practical point of view, one may also want to see not only *how much the solute signal can be enhanced* by RASER induction, but also *how strong this signal can be*. These two things are different as the former value is actually proportional to $\langle \hat{X}_z \rangle$ magnetization term (here "*X*" accounts for the solute spins), while the latter is a characteristic of $\langle \hat{X}_+ \rangle$ magnetization term. Since $\langle \hat{X} \rangle = \langle \hat{X}_z \rangle \cdot \sin\theta$, it is reasonable to add $SE_{mP,+}$ values to the analysis, $SE_{mP,+} = SE_{mP} \cdot \sin\theta$. The $SE_{mP,+}$ values are thus roughly proportional to the observed signal of a solute (while still corrected for the variations in HP hydrogenation product mP).

Section S4. Radiation damping measurements, calculation of ηQ product and threshold magnetization

Radiation damping measurements and ηQ product calculations for the NMR probe used in the 7.05 T NMR spectrometer were reported in the previous publication.[2] Below the same procedure is applied to the probe of the 1.4 T benchtop NMR spectrometer.

Radiation damping time τ_{RD} was measured according to previously established protocol. Full width at half-magnitude (FWHM) of the water signal was measured and used for the calculation using the following formula:[3]

$$\tau_{\mathrm{RD}} = \frac{0.8384}{\pi \cdot FWHM} \qquad (\mathrm{Eq.S1})$$

The sample of 90% H_2O / 10% D_2O mixture (in the standard 5 mm NMR tube) with a 0.6 mm OD catheter inside (similar to the sample composition in RASER experiments at 1.4 T spectrometer) had FWHM = 3.6 Hz, which resulted in $\tau_{\mathrm{RD}} = 74.1\ \mathrm{ms}$.

Further we use $\tau_{\mathrm{RD}} = \frac{74.1\ \mathrm{ms}}{2\pi\ \mathrm{rad}} = 11.8\ \frac{\mathrm{ms}}{\mathrm{rad}}$.

The product of the filling factor η and quality factor Q (ηQ) of the utilized probe may be determined using the following equation[4]:

$$\frac{1}{\tau_{\mathrm{RD}}} = \kappa\eta Q, \qquad \kappa = \frac{\mu_0\gamma_{\mathrm{H}} M_{\mathrm{z}}^{\mathrm{eq}}}{2} = \frac{\mu_0\gamma_{\mathrm{H}}^3\hbar^2 B_0 c_{\mathrm{A}}}{8k_{\mathrm{B}}T}\chi(\mathrm{H_2O}) \qquad (\mathrm{Eq.S2})$$

Necessary constants and parameters:

$\gamma_{\mathrm{H}} = 267.5\cdot 10^6\ \mathrm{rad\cdot s^{-1}\cdot T^{-1}}$ – the gyromagnetic ratio for protons;

$c_{\mathrm{A}} = (1.11\cdot 10^5\ \mathrm{mol\cdot m^{-3}})\cdot N_{\mathrm{A}} = 6.69\cdot 10^{28}\ \mathrm{m^{-3}}$ – the concentration of protons in pure water;

$\chi(\mathrm{H_2O}) = 0.90$ – molar fraction of light water in the sample;

$\mu_0 = 1.257\cdot 10^{-6}\ \mathrm{N\cdot A^{-2}}$ – the vacuum permeability;

$\hbar = \frac{h}{2\pi} = 1.055\cdot 10^{-34}\ \mathrm{J\cdot s\cdot rad^{-1}}$ – the reduced Planck's constant;

$k_{\mathrm{B}} = 1.381\cdot 10^{-23}\ \mathrm{J\cdot K^{-1}}$ – the Boltzmann's constant.

The measurement was conducted at $T = 299\ \mathrm{K}$ in magnetic field $B_0 = 1.4\ \mathrm{T}$, hence:

$$\eta Q = \frac{1}{\tau_{\mathrm{RD}}}\cdot\frac{8k_{\mathrm{B}}T}{\mu_0\gamma_{\mathrm{H}}^3\hbar^2 B_0 c_{\mathrm{A}}\chi(\mathrm{H_2O})};$$

$$\eta Q = \frac{1}{11.8\cdot 10^{-3}}\cdot\frac{8\cdot 1.381\cdot 10^{-23}\cdot 299}{1.257\cdot 10^{-6}\cdot(267.5\cdot 10^6)^3\cdot(1.055\cdot 10^{-34})^2\cdot 1.4\cdot 6.69\cdot 10^{28}\cdot 0.9}\cdot$$

$$\cdot\left(\frac{\mathrm{rad}}{\mathrm{s}}\cdot\frac{\mathrm{J\cdot K^{-1}\cdot K}}{\mathrm{N\cdot A^{-2}\cdot rad^3\cdot s^{-3}\cdot T^{-3}\cdot J^2\cdot s^2\cdot rad^{-2}\cdot T\cdot m^{-3}}}\right) = 124\left(\frac{1}{\mathrm{N\cdot A^{-2}\cdot T^{-2}\cdot J\cdot m^{-3}}}\right) =$$

$$= 124\left(\frac{1}{\mathrm{kg\cdot m\cdot s^{-2}\cdot A^{-2}\cdot kg^{-2}\cdot s^4\cdot A^2\cdot kg\cdot m^2\cdot s^{-2}\cdot m^{-3}}}\right) = 124.$$

Considering Eq. S2, we can now estimate initial magnetization M_0 of protons ^{1}H necessary for RASER in our system to initiate:

$$\frac{1}{\tau_{\mathrm{RD}}} = -\frac{\mu_0}{2}\eta Q\gamma_{\mathrm{H}} M_0 \rightarrow M_0 = -\frac{2}{\tau_{\mathrm{RD}}\mu_0\eta Q\gamma_{\mathrm{H}}};$$

$$M_0 = -\frac{2}{11.8\cdot 10^{-3}\cdot 1.257\cdot 10^{-6}\cdot 124\cdot 267.5\cdot 10^6}\cdot\left(\frac{\mathrm{rad}}{\mathrm{s\cdot kg\cdot m\cdot s^{-2}\cdot A^{-2}\cdot rad\cdot s^{-1}\cdot kg^{-1}\cdot s^2\cdot A}}\right) = -4.06\ \frac{\mathrm{mA}}{\mathrm{m}}.$$

Magnetization of protons in HP molecules (molar concentration c_{H} = 800 mM) can be recalculated to signal enhancement (*SE*). Below calculation is performed for a group containing only 1 proton:

$$SE_{\mathrm{crit}} = \frac{M_0}{M_{\mathrm{therm}}};\ M_{\mathrm{therm}} = \frac{(\gamma_{\mathrm{H}}\hbar)^2 B_0}{4k_{\mathrm{B}}T}\cdot c_{\mathrm{H}} N_{\mathrm{A}};$$

$$M_{\mathrm{therm}} = \frac{(267.5\cdot 10^6\cdot 1.055\cdot 10^{-34})^2\cdot 1.4}{4\cdot 1.381\cdot 10^{-23}\cdot 299}\cdot 800\cdot 6.023\cdot 10^{23}\cdot\left(\frac{\mathrm{rad^2\cdot s^{-2}\cdot T^{-2}\cdot J^2\cdot s^2\cdot rad^{-2}\cdot T}}{\mathrm{J\cdot K^{-1}\cdot K}}\cdot\mathrm{m^{-3}}\right) =$$

$$= 3.25\cdot 10^{-5}\ \mathrm{T^{-1}\cdot J\cdot m^{-3}} = 3.25\cdot 10^{-5}\ \mathrm{kg\cdot m^2\cdot s^{-2}\cdot kg^{-1}\cdot s^2\cdot A\cdot m^{-3}} = 0.0325\ \frac{\mathrm{mA}}{\mathrm{m}}.$$

$$SE_{\mathrm{crit}} = \frac{M_0}{M_{\mathrm{therm}}} = \frac{-4.06}{0.0325} \approx -125.$$

For benzene, which contains 6 equivalent protons, SE_{crit} is 6 times lower, $SE_{\mathrm{crit}} = -\frac{125}{6} = -21$.

Q factors of the NMR probes were measured from the respective wobble curves using the so-called "3 dB method" (see e.g. https://www.allaboutcircuits.com/textbook/alternating-current/chpt-6/q-and-bandwidth-resonant-circuit/). Wobble curve bandwidth ($\Delta\omega$) was measured at $H(1-1/\sqrt{2})$ distance from the dip of the wobble curve, where *H* is the full "height" of the wobble curve. This corresponds to the half power related to the maximum. Then *Q* factor was calculated as $Q = \frac{\omega_0}{\Delta\omega}$, where ω_0 is the tune frequency (at X-axis position of the wobble curve dip). The resultant *Q* factors were 720 for the probe of the 7.05 T spectrometer and 165 for the probe of the 1.4 T spectrometer.

Section S5. Simulations of RASER induction via PRINOE

A system of PHIP-produced polarization donor and a target solute (the NMR signal of which is enhanced via PRINOE) may be simplified as two strongly polarized protons *A*+*B* (polarization donor) and a separate ½-spin *X* (solute) interacting via cross-relaxation. The magnetization of these three spins also interacts with the RF coil of the NMR probe (Figure S3).

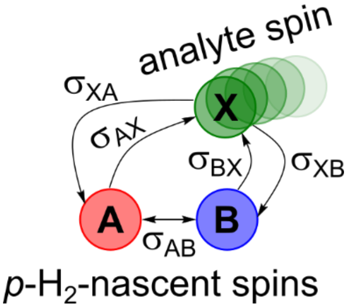

Figure S3. Schematic representation of the system used for simulations of PRINOE.

This system may then be described using modified Solomon equations:[2,5]

$$\begin{cases} \frac{d\langle\hat{A}_Z\rangle}{dt} = -(R_{1A}+\rho_A^*)(\langle\hat{A}_Z\rangle - A_{Z,eq}) - \sigma_{AB}(\langle\hat{B}_Z\rangle - B_{Z,eq}) - \sigma_{AX}(\langle\hat{X}_Z\rangle - X_{Z,eq}) + \Lambda_H\langle\hat{A}_+\rangle^2 \\ \frac{d\langle\hat{B}_Z\rangle}{dt} = -(R_{1B}+\rho_B^*)(\langle\hat{B}_Z\rangle - B_{Z,eq}) - \sigma_{BX}(\langle\hat{X}_Z\rangle - X_{Z,eq}) - \sigma_{BA}(\langle\hat{A}_Z\rangle - A_{Z,eq}) + \Lambda_H\langle\hat{B}_+\rangle^2 \\ \frac{d\langle\hat{X}_Z\rangle}{dt} = -(R_{1X}+\rho_X^*)(\langle\hat{X}_Z\rangle - X_{Z,eq}) - \sigma_{XA}(\langle\hat{A}_Z\rangle - A_{Z,eq}) - \sigma_{XB}(\langle\hat{B}_Z\rangle - B_{Z,eq}) + \Lambda_X\langle\hat{X}_+\rangle^2 \\ \frac{d\langle\hat{A}_+\rangle}{dt} = -i2\pi\nu_A\langle\hat{A}_+\rangle - R_{2A}\langle\hat{A}_+\rangle - \Lambda_H\langle\hat{A}_Z\rangle\langle\hat{A}_+\rangle \\ \frac{d\langle\hat{B}_+\rangle}{dt} = -i2\pi\nu_B\langle\hat{B}_+\rangle - R_{2B}\langle\hat{B}_+\rangle - \Lambda_H\langle\hat{B}_Z\rangle\langle\hat{B}_+\rangle \\ \frac{d\langle\hat{X}_+\rangle}{dt} = -i2\pi\nu_X\langle\hat{X}_+\rangle - R_{2X}\langle\hat{X}_+\rangle - \Lambda_X\langle\hat{X}_Z\rangle\langle\hat{X}_+\rangle \end{cases} \quad \text{(Eq. S3)}$$

where the variables are defined as follows:

- $\langle\hat{A}_Z\rangle$, $\langle\hat{B}_Z\rangle$ and $\langle\hat{X}_Z\rangle$ are the longitudinal molar polarizations of the spins *A*, *B* and *X*, respectively;
- $\langle\hat{A}_+\rangle$, $\langle\hat{B}_+\rangle$ and $\langle\hat{X}_+\rangle$ are the transverse molar polarizations of the spins *A*, *B* and *X*, respectively;
- $A_{Z,eq}$, $B_{Z,eq}$ and $X_{Z,eq}$ are the corresponding longitudinal molar polarizations at thermal equilibrium;
- R_{1i} are the spin-lattice relaxation rates ($1/T_{1,i}$);
- R_{2i} are the spin-spin relaxation rates ($1/T_{2,i}$);
- ρ_i^* are the sums of relevant intra- and intermolecular cross-relaxation rates σ_{ii} (diagonal elements in matrix representation of Solomon equations);
- σ_{ij} are the intra- and intermolecular cross-relaxation rates between unlike nuclear spins ($\sigma_{AB} = \sigma_{BA}$);
- ν_i are the offsets of the Larmor frequency (Hz) from the frequency corresponding to 0 ppm of the corresponding nuclei;
- Λ_i are the constants derived from the RASER equations for molar polarizations (see the derivation below).

RASER threshold is given by the following equation:[6]

$$\frac{1}{\tau_{RD}} = -\frac{\mu_0}{2}\eta Q\gamma M_0 = -\frac{\mu_0}{4}\eta Q\gamma^2\hbar n_S P_0 \Rightarrow \frac{1}{M_0\tau_{RD}} = -\frac{\mu_0\eta Q\gamma}{2}$$

where μ_0 is vacuum permeability, η is the coil filling factor, *Q* is the resonator quality factor, γ is the gyromagnetic ratio, M_0 is the initial magnetization, $\hbar$ is the reduced Planck constant, n_S is the spin density, and P_0 is the initial nuclear spin polarization.

Usually, the radiation damping component for spin I that leads to RASER is defined as $\frac{\langle\hat{I}_+\rangle^2}{|M_0\tau_{RD}|}$ or $\frac{\langle\hat{I}_Z\rangle\langle\hat{I}_+\rangle}{|M_0\tau_{RD}|}$.[7] There is a conceptual problem with such definition, which is the fact that initial magnetization of the entire sample is net zero while of individual groups of spins it is non-zero. To remedy this, let us recombine constants taking molar polarization M_{mp} as a primary measure of sample's magnetization:

$$\frac{\mu_0}{2}\eta Q\gamma M_0 = \lambda M_0 = \lambda n_S m_I\gamma\hbar P_0 = \frac{1}{2}\lambda\frac{N}{V}\gamma\hbar P_Z = \frac{1}{2}N_A\gamma\hbar\lambda\cdot\frac{N}{VN_A}P_Z$$

$$\Lambda = \frac{1}{2}N_A\gamma\hbar\lambda = \frac{\mu_0}{4}\eta Q\gamma^2 N_A\hbar$$

$$M_{mp} = \frac{N}{VN_A}P_Z = P_Z\cdot C$$

where C – concentration in mol/m^3 = mmol/L.

$$[M_{mp}] = \frac{\text{mol}}{\text{m}^3}$$

$$[\Lambda] = [N_A\gamma\hbar\lambda] = \left[\frac{1}{\text{mol}}\right][\text{A}\cdot\text{m}^2]\left[\frac{\text{rad}\cdot\text{m}}{\text{s}\cdot\text{A}}\right] = \left[\frac{\text{rad}\cdot\text{m}^3}{\text{mol}\cdot\text{s}}\right]$$

Hence, M_{mp} is molar polarization in mmol/L or mol/m^3, and Λ is corresponding term for introduction of radiation damping.

The intramolecular cross-relaxation rates $\sigma_{ij,\mathrm{intra}}$ for unlike spins are given as:[8]

$$\sigma_{ij,\mathrm{intra,unlike}} = n_i\left(W_2^{ij} - W_0^{ij}\right),$$
$$\sigma_{ji,\mathrm{intra,unlike}} = n_j\left(W_2^{ji} - W_0^{ji}\right),$$

where the transition probabilities for spins-½ are given by

$$W_0^{ij} = \frac{1}{10}\left(\frac{\mu_0\gamma_i\gamma_j\hbar}{4\pi d_{ij}^3}\right)^2 \times J_{ij}\left(\omega_i - \omega_j\right),$$
$$W_2^{ij} = \frac{6}{10}\left(\frac{\mu_0\gamma_i\gamma_j\hbar}{4\pi d_{ij}^3}\right)^2 \times J_{ij}\left(\omega_i + \omega_j\right),$$

where d_{ij} is the fixed distance between nuclei *i* and *j* (specifically, d_{AB} = 2.4 Å). Spectral density function $J_{ij}(\omega)$ is given as

$$J_{ij}(\omega) = \frac{\tau_{\mathrm{c},ij}}{1 + \left(\omega\tau_{\mathrm{c},ij}\right)^2},$$

where $\tau_{\mathrm{c},ij}$ is the correlation time for interactions between nuclei *i* and *j*.

For intermolecular cross-relaxation rates $\sigma_{ij,\mathrm{inter}}$, the definitions are:[9]

$$\sigma_{ii,\mathrm{inter,like}} = \frac{8\pi}{15} I_i(I_i + 1)\gamma_i^4\hbar^2 \frac{N_j}{d_{ii}D_{ii}}\left(\frac{\mu_0}{4\pi}\right)^2,$$
$$\sigma_{ii,\mathrm{inter,unlike}} = \frac{16\pi}{45} I_j\left(I_j + 1\right)\gamma_i^2\gamma_j^2\hbar^2 \frac{N_j}{d_{ij}D_{ij}}\left(\frac{\mu_0}{4\pi}\right)^2,$$
$$\sigma_{jj,\mathrm{inter,unlike}} = \frac{16\pi}{45} I_i(I_i + 1)\gamma_i^2\gamma_j^2\hbar^2 \frac{N_i}{d_{ij}D_{ij}}\left(\frac{\mu_0}{4\pi}\right)^2,$$
$$\sigma_{ij,\mathrm{inter,unlike}} = \frac{8\pi}{45} I_i(I_i + 1)\gamma_i^2\gamma_j^2\hbar^2 \frac{N_i}{d_{ij}D_{ij}}\left(\frac{\mu_0}{4\pi}\right)^2,$$
$$\sigma_{ji,\mathrm{inter,unlike}} = \frac{8\pi}{45} I_j\left(I_j + 1\right)\gamma_i^2\gamma_j^2\hbar^2 \frac{N_j}{d_{ij}D_{ij}}\left(\frac{\mu_0}{4\pi}\right)^2,$$

where I is the spin of the corresponding nucleus, *N* is the concentration of nuclei (m^{-3}), D_{ij} is the mutual self-diffusion constant defined via individual self-diffusion coefficients as $1/2\left(D_i + D_j\right)$, and d_{ij} is the distance of closest approach for atoms *i* and *j* (assessed as van der Waals radius of ^{1}H nucleus ~ 1.2 Å).

The following simplifications are made in this model:

1. No *J*-coupling interactions are taken into account between spins *A* and *B*.
2. Initial spin order is considered to be $|A_Z\rangle - |B_Z\rangle$, although in ALTADENA conditions the $|A_Z\rangle - |B_Z\rangle - 2|A_ZB_Z\rangle$ spin order is in fact generated after adiabatic transfer into the NMR probe.[10] No evolution of the nascent two-spin order component $|A_ZB_Z\rangle$ is taken into account.
3. Continuous hydrogenation of the precursor molecules inside the NMR probe (at high-field, or PASADENA conditions) is not taken into account. The reason is that, as in the simplification 2, no evolution of the two-spin order component $|A_ZB_Z\rangle$ that is produced in PASADENA conditions[11] is simulated.

First, it was tested that a pair of ALTADENA-hyperpolarized spins *A* and *B* may induce RASER of the third spin *X* present in the system via intermolecular NOE interactions. Initial *SE* for spins *A* and *B* was assumed to be ±7000 as it corresponds to experimentally observed initial $P_{1\mathrm{H}}$ ~ 17% in HP **1**. Small 1° flips onto magnetizations were applied every 1 s, and between the flips evolution of magnetizations according to the equations introduced above was allowed. As a result, RASER signal of *X*, affected by regular flips was obtained (Figure 2f–h in the main text).

Following this, series of simulations with varied flip angles *θ* and interpulse delays t_R were conducted. The heatmaps were simulated with different degree of detail, demonstrating complex dependence of NMR signal enhancement of spin *X* on the pulsing parameters. In general, decrease of *SE* is observed with the increase of *θ* and t_R, however, this dependence is non-monotonous (Figure S4). While the simulated 4×4 heatmap generally is in agreement with the experimental heatmap, which has the same resolution (Figure S4a), the comparison of the experiment with simulated heatmaps with higher level of detalization is not straightforward (Figure S4b and S4c).

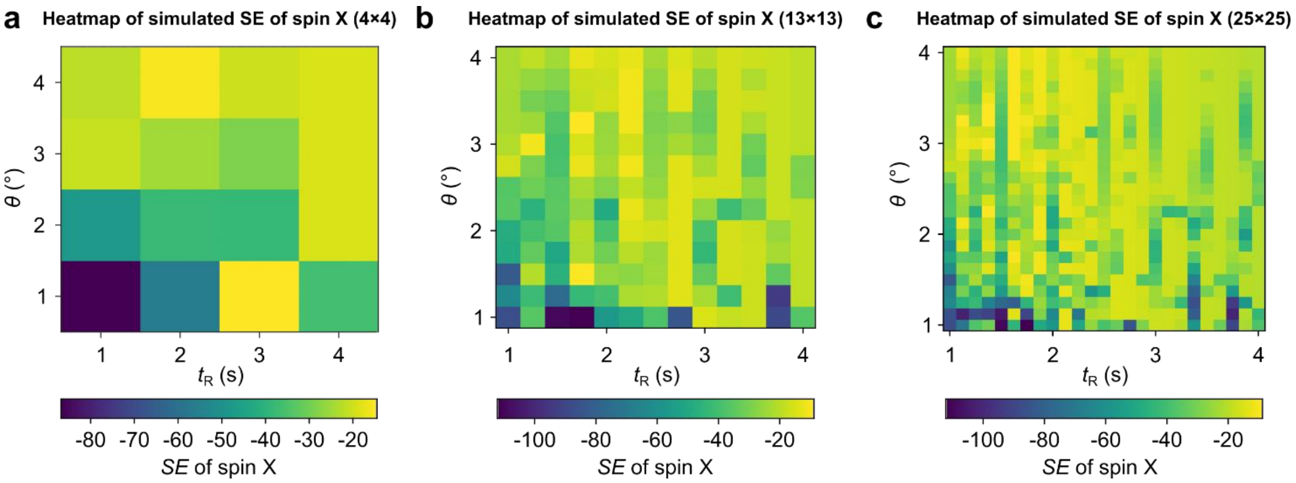

Figure S4. Heatmaps of *SE* of spin *X* achieved in the PRINOE simulations with varying θ and t_R parameters with different resolutions across the axes: (**a**) 4×4, (**b**) 13×13 (169 simulations), and (**c**) 25×25 (625 simulations). Conducting more detailed simulations would be very time consuming, as each point requires ca. 3–4 minutes, hence, several weeks of simulation time would be required to significantly improve the resolution. Note that in the simulations *SE* and *SE/mP* are only different by a certain scaling factor as initial molar polarization of spins *A* and *B* does not vary between different simulation runs.

Varying the t_R delay between the applied magnetization flips in simulations revealed an expected trend of biexponential PRINOE-like dependence. However, at short t_R values several outlier points for this trend were obtained, especially in the range of t_R = 1–10 s (Figure S5).

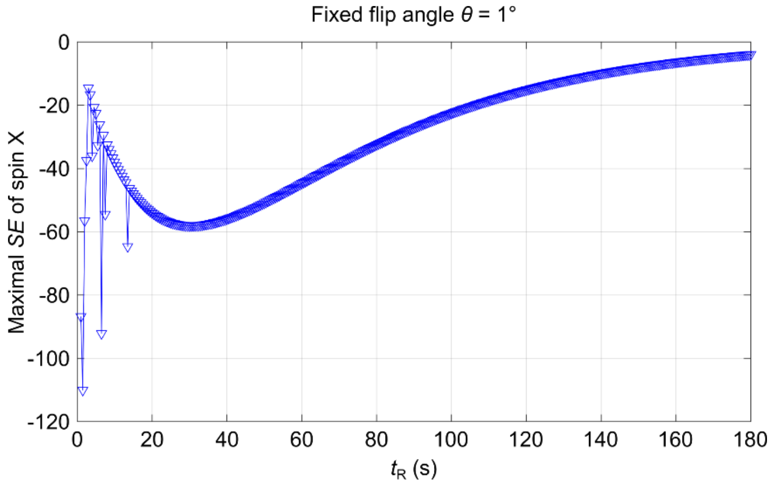

Figure S5. Simulated dependence of maximal achievable SE of spin X on the interpulse delay t_R with a constant flip angle of 1°. Delays from 1 to 180 s with a step of 0.5 s were implemented.

Increasing the flip angle with a fixed short interpulse delay t_R leads to decreasing of observable *SE*, although the dependence of the normalized $SE_{mP}\cdot\sin\theta$ is more complex (Figure S6).

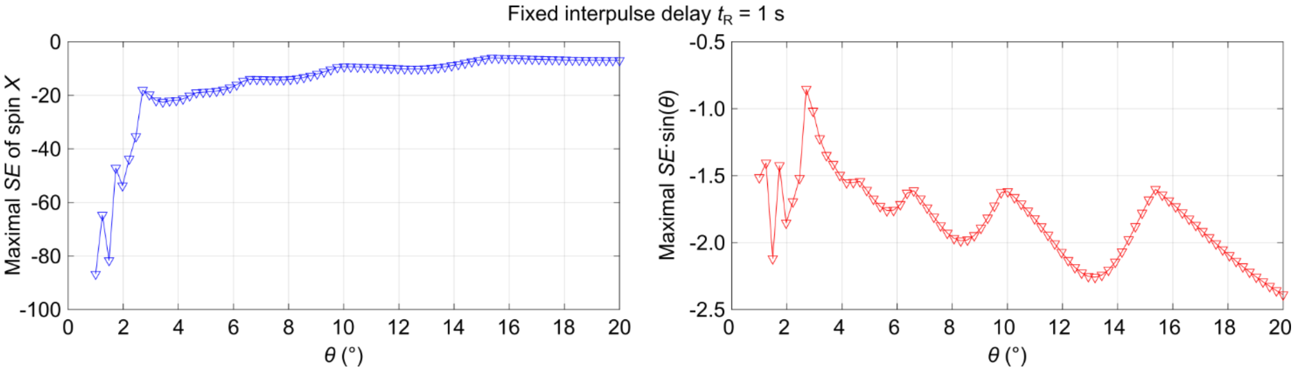

Figure S6. (**a**) Simulated dependence of maximal achievable *SE* of spin *X* on the flip angle θ with a constant t_R of 1 s. Flip angles from 1° to 20° with a step of 0.25° were implemented. (**b**) Simulated dependence of maximal achievable $SE\cdot\sin\theta$ of spin *X* on the flip angle θ with a constant t_R of 1 s. Flip angles from 1° to 20° with a step of 0.25° were implemented.

Overall, the above simulations demonstrate that utilizing frequently applied small flip angle RF pulses is the best way to trigger RASER of the solute via PRINOE, confirming the experimental observations.

Section S6. ^{1}H RASER of benzene triggered by a single RF pulse

Flip angles of 1, 3, 6, 10, 15, 20, 30, 90° were tested. For a 10° pulse an additional experiment with a shorter 42 s delay before the RF pulse application (instead of typical 64 s delay) was performed to show that no RASER is observed when PRINOE signal is not high enough. Furthermore, an experiment without RF pulse application was performed to demonstrate that at these conditions RASER is not induced as well. The corresponding spectrographs are presented in Table S4. Note that ^{1}H NMR signals at ~7.5 ppm correspond to benzene, while signals at ~4.3 and ~5.3 ppm to *1d* and *1b* protons of **1**, respectively.

Table S4. ^{1}H NMR spectrographs of RASER signals obtained after application of a single RF pulse.

<table>
<tr><th>Flip angle, °</th><th>RASER signals and corresponding spectrographs[a]</th></tr>
<tr><td>no RF pulse</td><td>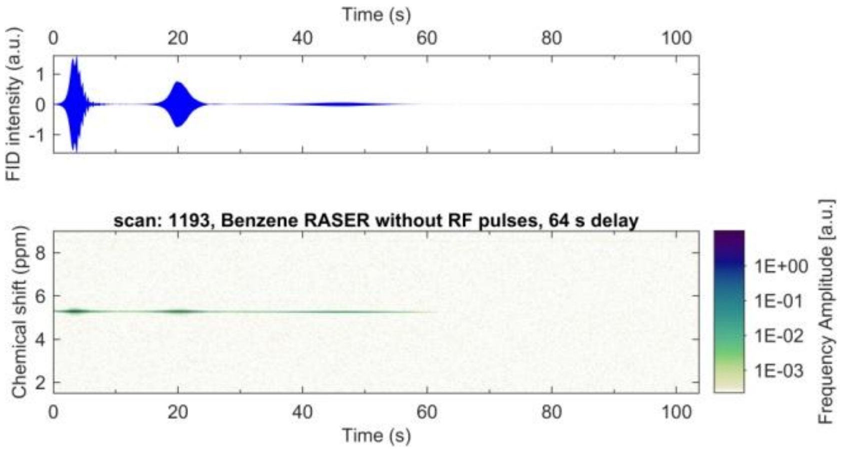
</td></tr>
<tr><td>1</td><td>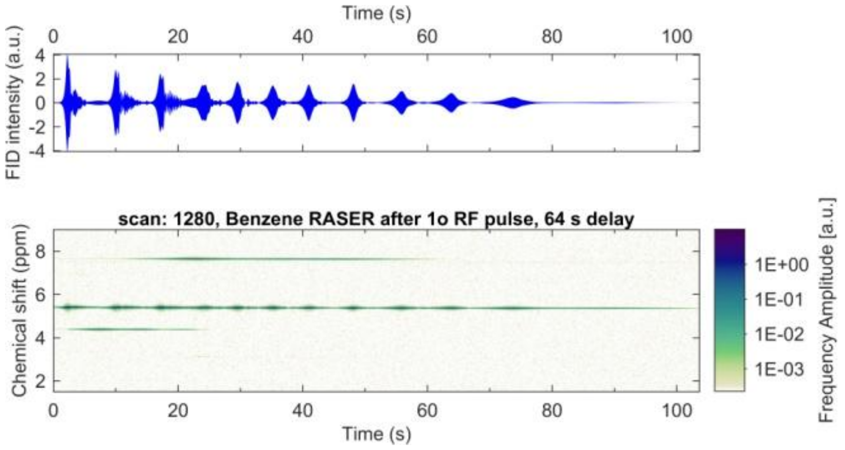
</td></tr>
<tr><td>3</td><td>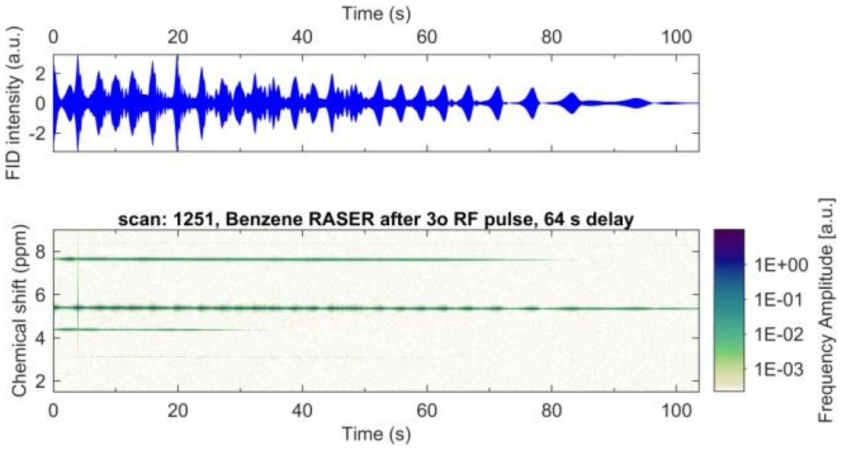
</td></tr>
</table>

6	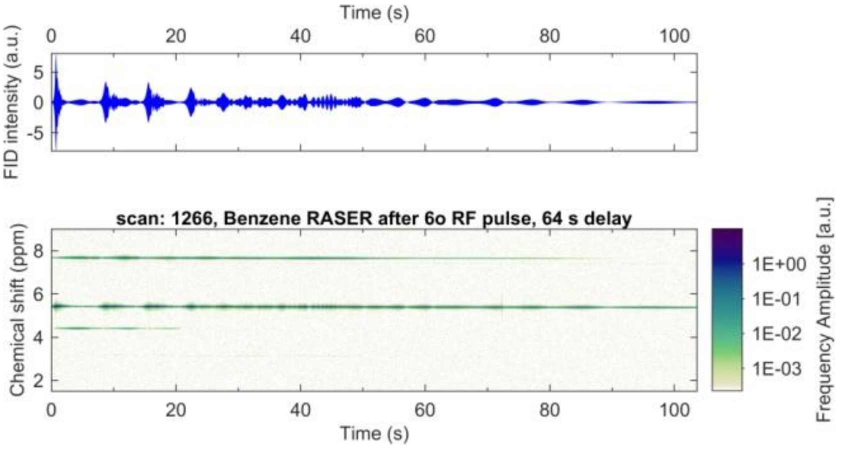
10<br>(64 s delay)	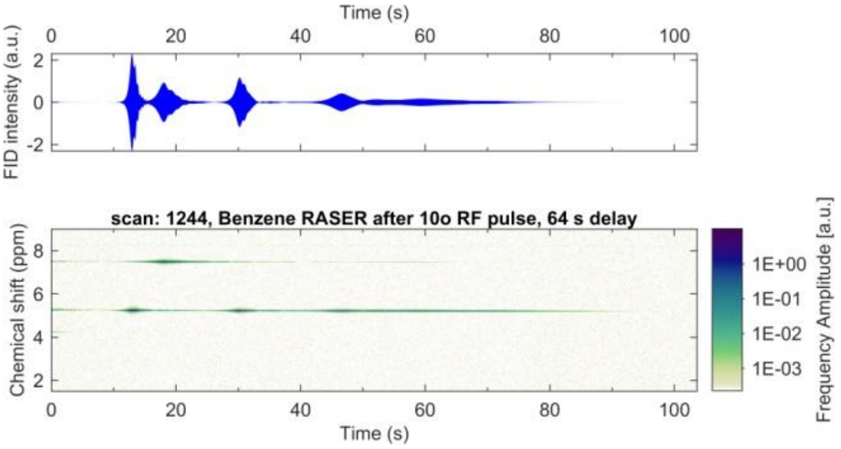
10<br>(42 s delay)	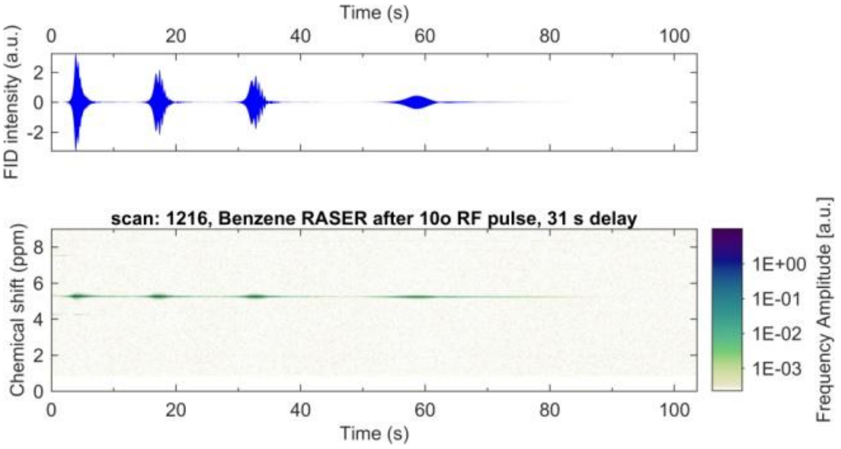
15	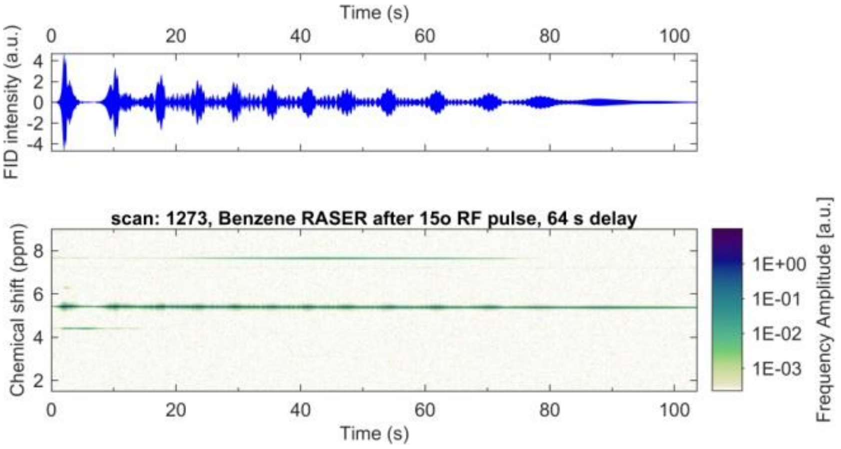

20	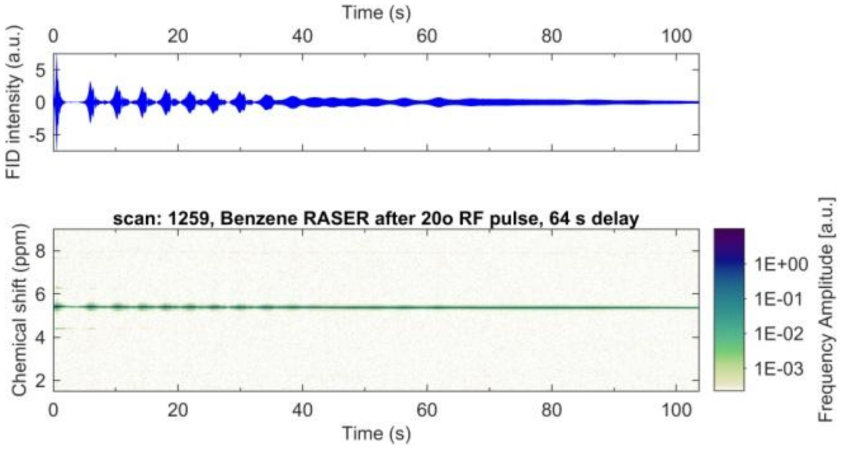
30	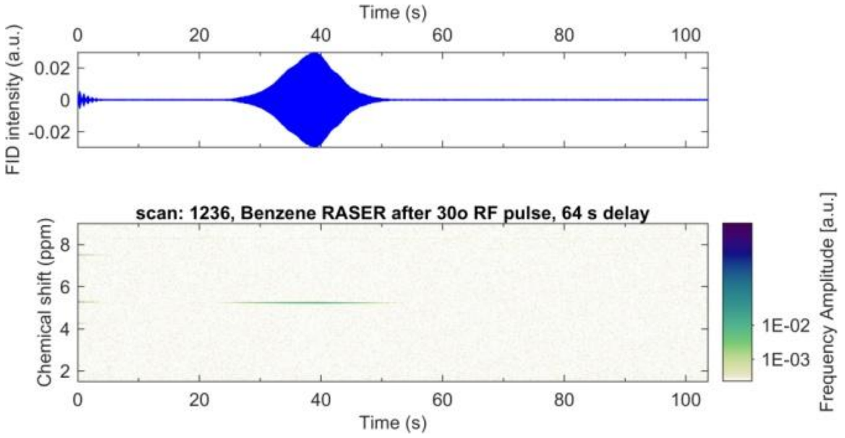
90	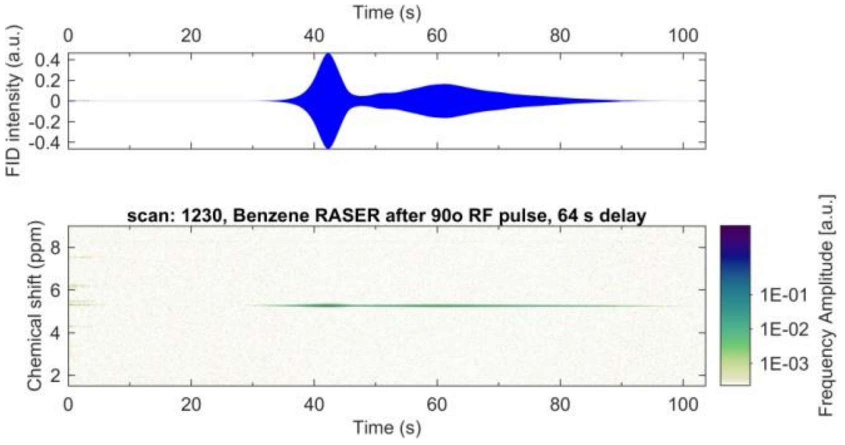

[a] The FID intensities (in RASER signal evolution graphs) and frequency amplitudes (in the corresponding spectrographs) are consistent for all figures presented here.

More or less reproducible trend was observed: low flip angles (1–10°) provided strong RASER, high flip angles (15–90°) provided very weak RASER of benzene (see Figure S7). The reason is likely that RF pulses with higher flip angle (>10°) deplete polarization of HP ^{1}H nuclei, and residual magnetization of solute is then not enough to invigorate RASER activity. Also note that it is difficult to directly compare intensities in these single-pulse experiments, as RASER signal manifests itself as oscillations in the NMR spectra.

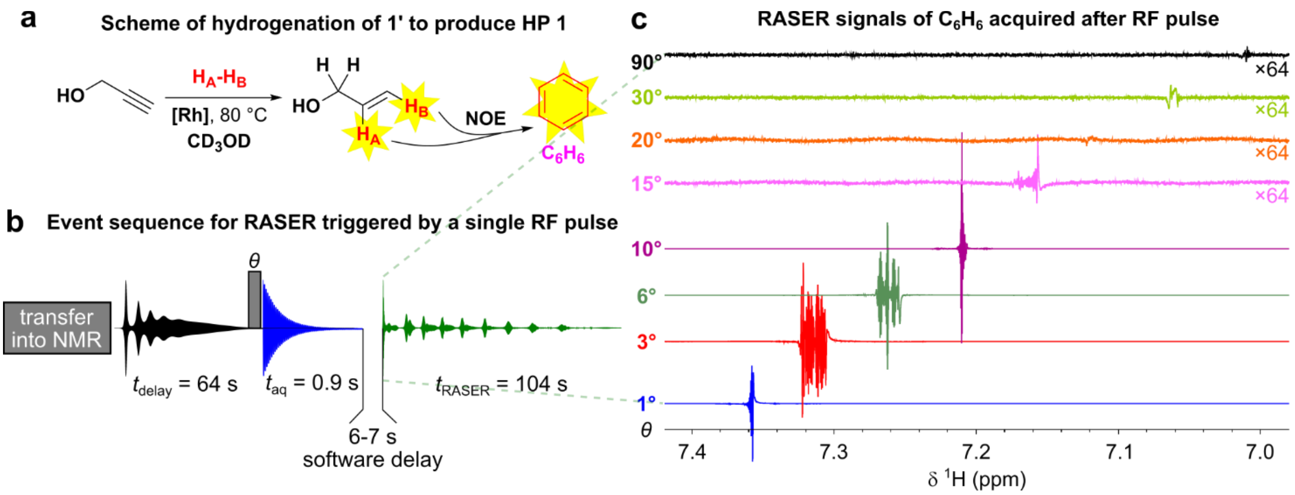

Figure S7. (**a**) Reaction scheme of pairwise addition of *p*-H_2 to **1'** yielding HP **1** with subsequent transfer of polarization to benzene via NOE. (**b**) Schematic event sequence in experiments with benzene RASER triggering by a single RF pulse. (**c**) Benzene regions of ^{1}H NMR spectra produced by FT of the subsequent RASER signal. The pulse flip angle θ increases from bottom to top: 1°, 3°, 6°, 10°, 15°, 20°, 30°, 90°. The top four spectra are scaled by a factor of ×64, and all spectra are also shifted along the horizontal axis with 0.1 ppm increment for better representation.

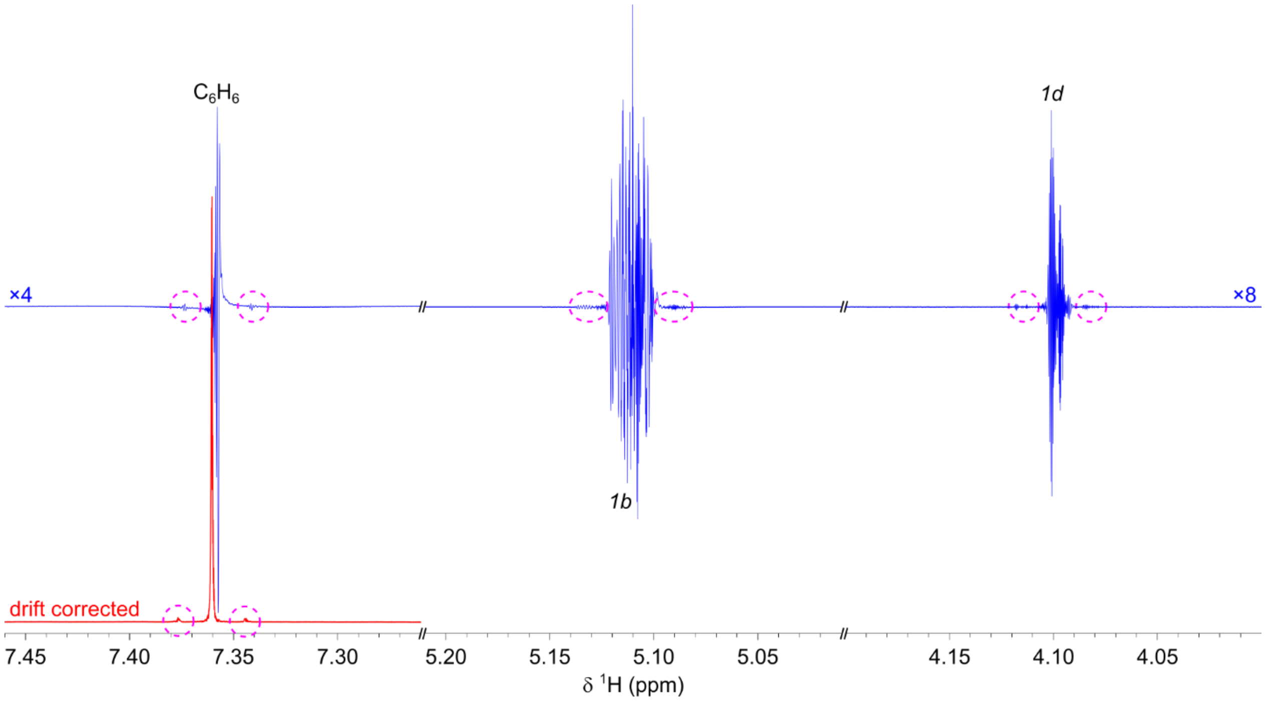

Figure S8. ^{1}H NMR spectrum acquired after application of 1° RF pulse exhibiting RASER signals of *1b*, *1d* and C_6H_6 and satellite signals (marked with purple circles). Note that signals of *1d* and C_6H_6 are zoomed to better show the accompanying artifacts. The artifacts also get narrowed after field drift correction as highlighted in the corrected signal of C_6H_6 (red).

Section S7. ¹H RASER of benzene triggered by RF pulse trains

Experiments with detection of RASER triggered by trains of 1° RF pulses applied with 1 s interpulse delay (similar to the one presented in Figure 2c,d, with the exception of θ and t_R) were conducted using benzene solute and HP donors **1**, **3**, and **4**. Durations of pulse trains were 58–64 s for substrate **1**, 59–61 s for **3**, and 54–55 s for **4**. The obtained spectrographs are presented in Table S5. Note that ^{1}H NMR signals at ~7.5 ppm correspond to benzene, and other signals in the range of 5–6 ppm correspond to the *b* protons of **1**, **3** or **4**.

Table S5. ^{1}H NMR spectrographs of RASER signals obtained after application of 1°/1 s RF pulse trains.

HP donor	RASER signals and corresponding spectrographs
**1**	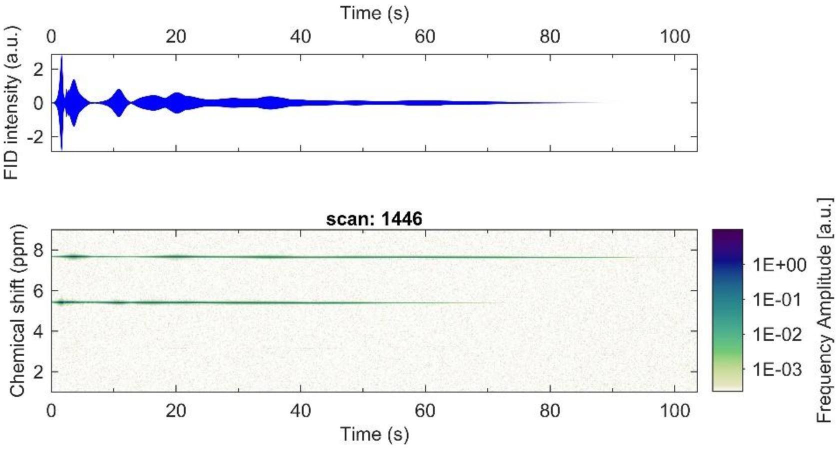
**1**	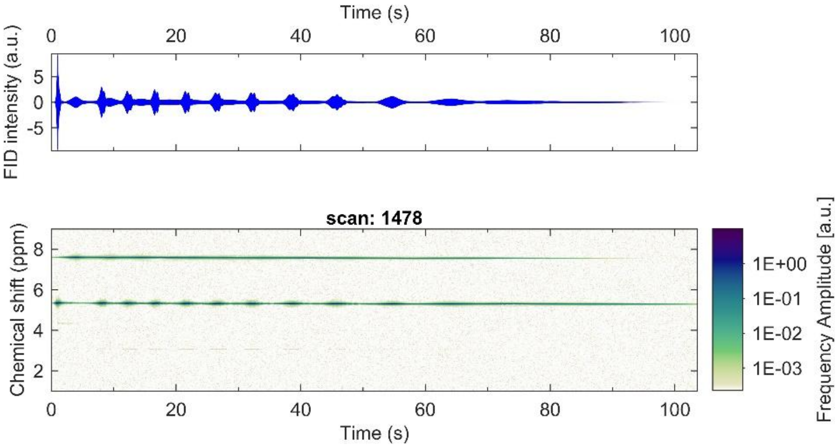
**3**	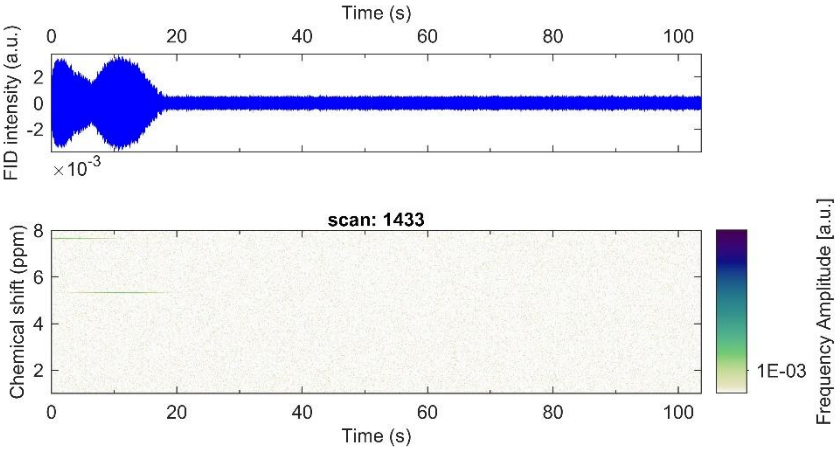

**3**	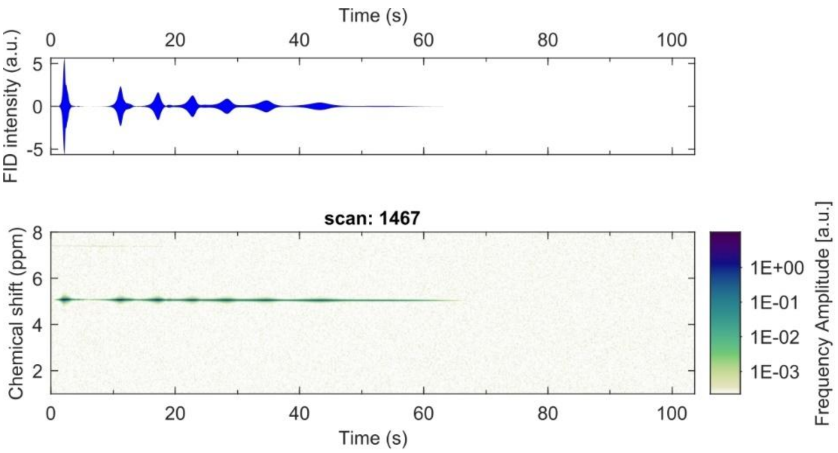
**3**	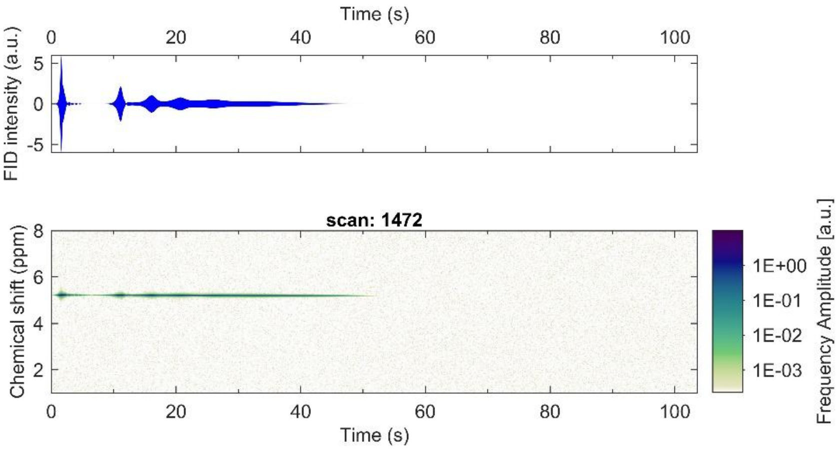
**4**	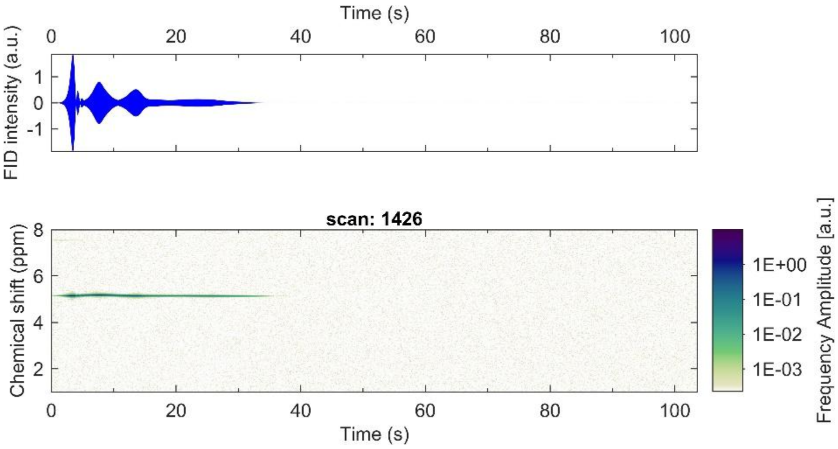
**4**	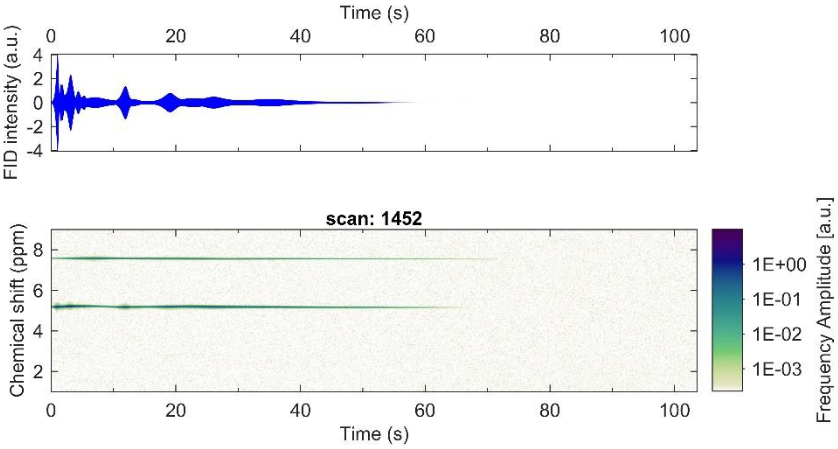

Section S8. Comparative efficiency of hyperpolarized donors 1–4 for PRINOE and RASER induction

Patterns of the decay of the ^{1}H polarization of the HP products **1**–**4** are similar (Figure S9c,f,i,l). It is important to note that polarization of both added H_A and H_B atoms is positive, while typically in ALTADENA experiments protons H_B should have negative polarization. This inversion of polarization may be attributed to combination of RASER and application of RF pulses–initial RASER burst on H_B protons flips negative polarization to positive and further application of RF pulses prevents it from flipping back to negative. The RASER bursts in the beginning of acquisition were evident in the first spectra obtained after the samples were placed inside the NMR probe. Positive polarization of other *J*-coupled protons is then rapidly suppressed to near-zero or negative values, most likely, due to NOE interactions with the positively polarized H_A and H_B nuclei (such biexponential NOE curve is best visible for the *c* protons in the graphs in Figure S9).

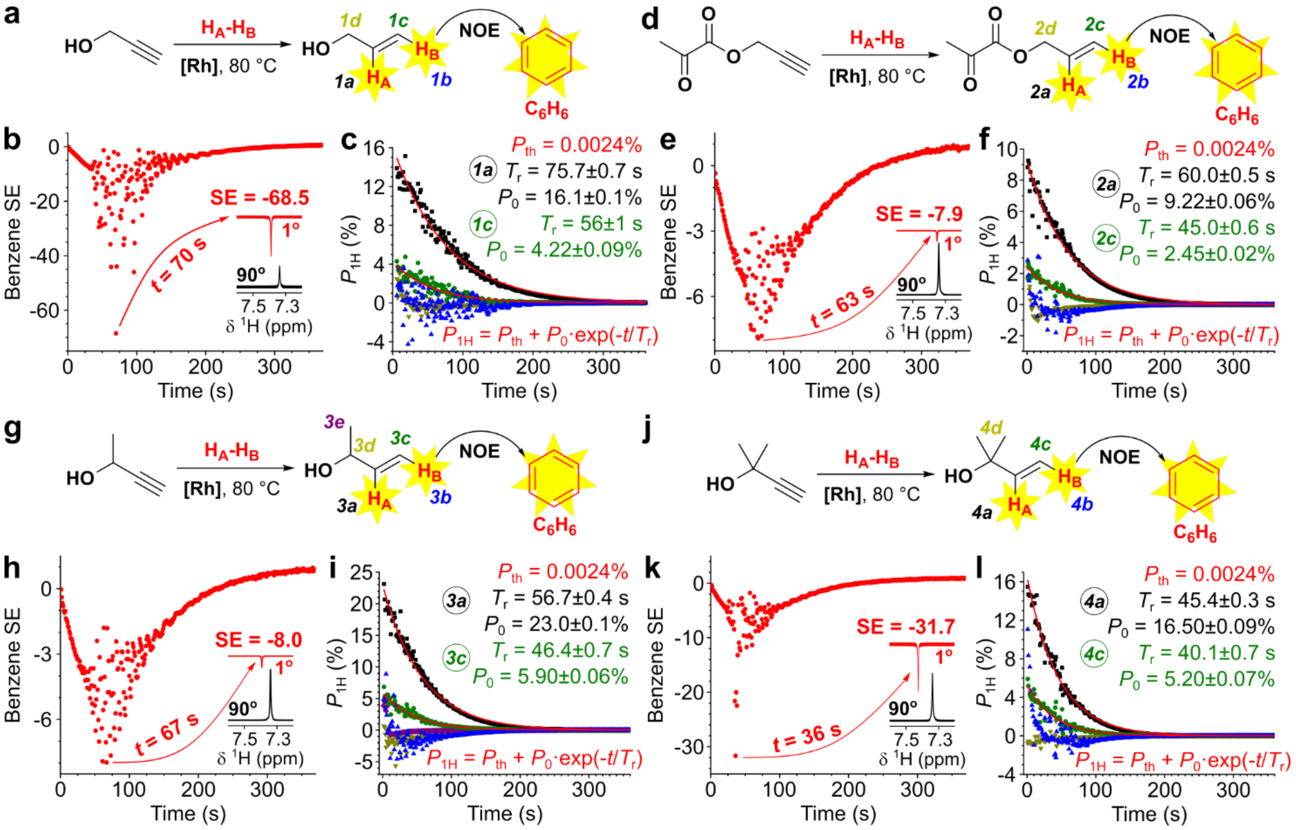

Figure S9. (**a**) Reaction scheme of pairwise addition of *p*-H_2 to **1'** yielding HP **1**. (**b**) Evolution of ^{1}H NMR signal enhancement (*SE*) of benzene using HP **1** as a polarization donor. Inset: regions of ^{1}H NMR spectra presenting the most enhanced (red, 1° flip pulse) and thermal (black, 90° flip pulse) signal of benzene. (**c**) Evolution of ^{1}H polarization (P_{1H}) of **1** during the PRINOE experiment, fitting equation and corresponding fitting parameters. (**d**) Reaction scheme of pairwise addition of *p*-H_2 to **2'** yielding HP **2**. (**e**) Evolution of ^{1}H NMR *SE* of benzene using HP **2** as a polarization donor. Inset: regions of ^{1}H NMR spectra presenting the most enhanced (red, 1° flip pulse) and thermal (black, 90° flip pulse) signal of benzene. (**f**) Evolution of P_{1H} of **2** during the PRINOE experiment, fitting equation and corresponding fitting parameters. (**g**) Reaction scheme of pairwise addition of *p*-H_2 to **3'** yielding HP **3**. (**h**) Evolution of ^{1}H NMR *SE* of benzene using HP **3** as a polarization donor. Inset: regions of ^{1}H NMR spectra presenting the most enhanced (red, 1° flip pulse) and thermal (black, 90° flip pulse) signal of benzene. (**i**) Evolution of P_{1H} of **3** during the PRINOE experiment, fitting equation and corresponding fitting parameters. (**j**) Reaction scheme of pairwise addition of *p*-H_2 to **4'** yielding HP **4**. (**k**) Evolution of ^{1}H NMR *SE* of benzene using HP **4** as a polarization donor. Inset: regions of ^{1}H NMR spectra presenting the most enhanced (red, 1° flip pulse) and thermal (black, 90° flip pulse) signal of benzene. (**l**) Evolution of P_{1H} of **4** during the PRINOE experiment, fitting equation and corresponding fitting parameters. In panels (a,d,g,j), groups of protons in the molecules **1**–**4** are marked with the same colors as in respective panels (c,f,i,l) where evolution of P_{1H} of these protons is presented. In panels (b,e,h,k) the presented data were obtained with individual phase correction of each 1D NMR spectrum in a pseudo-2D set so that the NMR signal of benzene becomes purely emissive. In panels (c,f,i,l) several strongly outlier data points were omitted due to strong RASER influence.

Inferior efficiency of **3** and **4** as HP donors may be attributed to the fact that they contain additional groups that receive polarization via intramolecular PRINOE that is more efficient than intermolecular polarization transfer to benzene. Indeed, RASERs of **3e** (induced via intramolecular *J*-couplings, but PRINOE contribution is possible as well) and **4d** (induced via intramolecular PRINOE) protons were often observed. Relevant examples of these phenomena are provided in Figure S10.

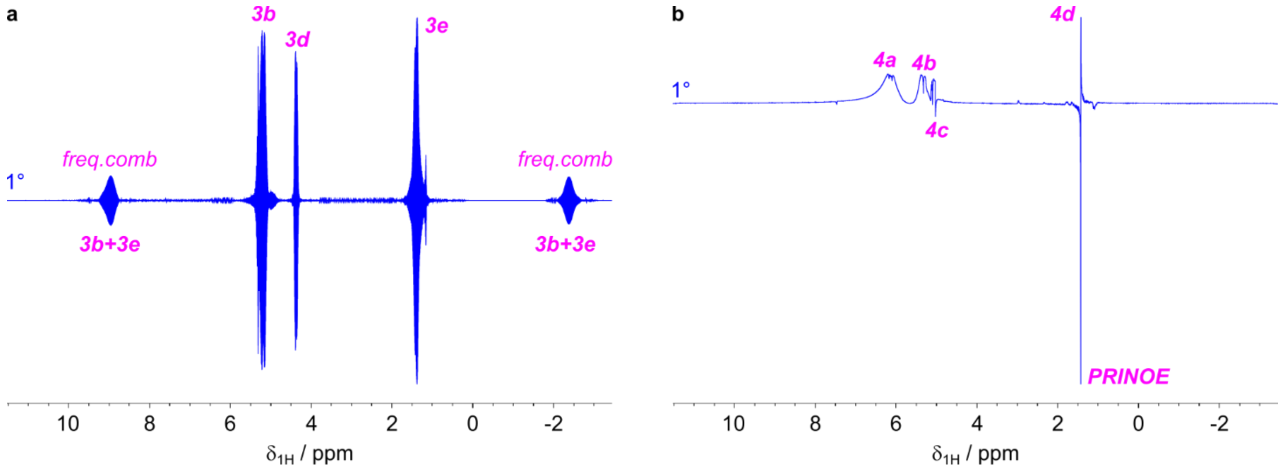

Figure S10. The ^{1}H NMR spectra from the pseudo-2D series demonstrating RASER activity of (**a**) the **3e** (1st in the series) and (**b**) **4d** protons (7th in the series).

Table S6. Average chemical conversion of **1'–4'** to **1–4** (*X*), initial molar polarization of HP donors **1–4** and benzene (*mP*), maximum ^{1}H NMR signal enhancement factors of benzene (*SE*), fraction of achieved molar polarization of benzene and HP donors (|*mP*(C_6H_6)/*mP*(donor)|), and maximum normalized signal enhancement factors (*SE*/*mP*) obtained in PAINTER experiments employing RF pulse trains with a flip angle of 1° and repetition time of 1 s. Note that *SE* and *SE*/*mP* values were obtained using averaging of 10 maximal data points in each experiment; the detailed calculation procedure can be found in Section S3. Although *X* and *mP* values should not depend on RF pulsing protocol, for **1** here we used only data from the experiments with 1°/1 s pulse trains for a consistent comparison with other HP donors.

HP donor	*X*, %	*mP*(donor), mM	*mP*(C_6H_6), mM	|*mP*(C_6H_6)/*mP*(donor)|	*SE*(C_6H_6)	*SE*(C_6H_6)/*mP*(donor), M^{-1}
**1**	64 ± 4	101 ± 1	–0.82±0.20	≈8.1·10^{-3}	–42 ± 11	–420 ± 105
**2**	63 ± 1	75 ± 3	–0.12±0.02	≈1.6·10^{-3}	–6 ± 1	–82 ± 13
**3**	44 ± 2	76 ± 8	–0.12±0.02	≈1.6·10^{-3}	–6.3 ± 0.8	–84 ± 16
**4**	60 ± 11	107 ± 13	–0.3±0.1	≈2.5·10^{-3}	–14 ± 5	–125 ± 41

Section S9. PRINOE studies of other solutes beyond benzene

The following solutes were tested using 1°/1 s RF pulse train protocol and **1** as an HP donor: furan, which demonstrated strong PRINOE in the previous work[12], several biologically relevant molecules (ethyl pyruvate, methyl (*S*)-lactate, pyruvic acid, glycine, choline chloride), acetonitrile, and several molecules with high payload of chemically equivalent protons (urotropine, cyclohexane, hexamethyldisiloxane).

The following compounds did not show any PRINOE enhancement at all as a result of dramatically reduced catalytic activity, likely caused by coordination of these molecules to Rh catalyst: methyl (*S*)-lactate, acetonitrile, pyruvic acid, urotropin, glycine, choline chloride. In principle, all of these compounds may also be potentially employed in PRINOE experiments, if one injects them into solution after PHIP experiment when the HP molecules **1**–**4** have been already produced. However, this would also lead to shorter RASER activity as a result of sample depressurization and contact with oxygen, accelerating the relaxation of ^{1}H polarization.

Furan and EtPyr yielded PRINOE effects but without RASER (Figure S11a,b). Furan has two groups of inequivalent protons and there are 3 times less of equivalent protons than for benzene, hence their molar polarization apparently is not strong enough to induce RASER (despite quite good signal enhancement).

CyH clearly showed PRINOE oscillations serving as a proof of RASER (Figure S11c). It should be acknowledged that cyclohexane signal overlaps with the signal of a by-product formed from **1'** (which we assign as 1-methoxypropene), which has shown RASER effects in some of the experiments where benzene solute was used rather than CyH. However, RASER of 1-methoxypropene is observed only in the very beginning of the pseudo-2D dataset, while CyH RASER typically emerges later. From the three experiments performed with CyH, only one showed some signs of possible interference from 1-methoxypropene RASER. In this case, the averaged RASER signals of 1-methoxypropene (from benzene data) were subtracted from the observed signals from CyH data, and the resulting difference was assessed.

HMDSO showed lower *SE* than furan, but it demonstrated oscillations (i.e., RASER) in some of the experiments because of the higher 18 protons payload (Figure S11d). However, reproducibility of results with HMDSO was low: in some experiments it showed PRINOE with RASER, in other experiments – PRINOE without RASER. The corresponding HMDSO data were averaged and evaluated separately to clearly demonstrate this difference in Table 1. Thus, it seems that signal intensity of HP HMDSO is close to the RASER threshold and, thus, the observation of PRINOE oscillations (i.e., RASER) strongly depends on homogeneity of the spectrometer field, variations of **1'** conversion and polarization of **1**, chaoticity of RASER etc.

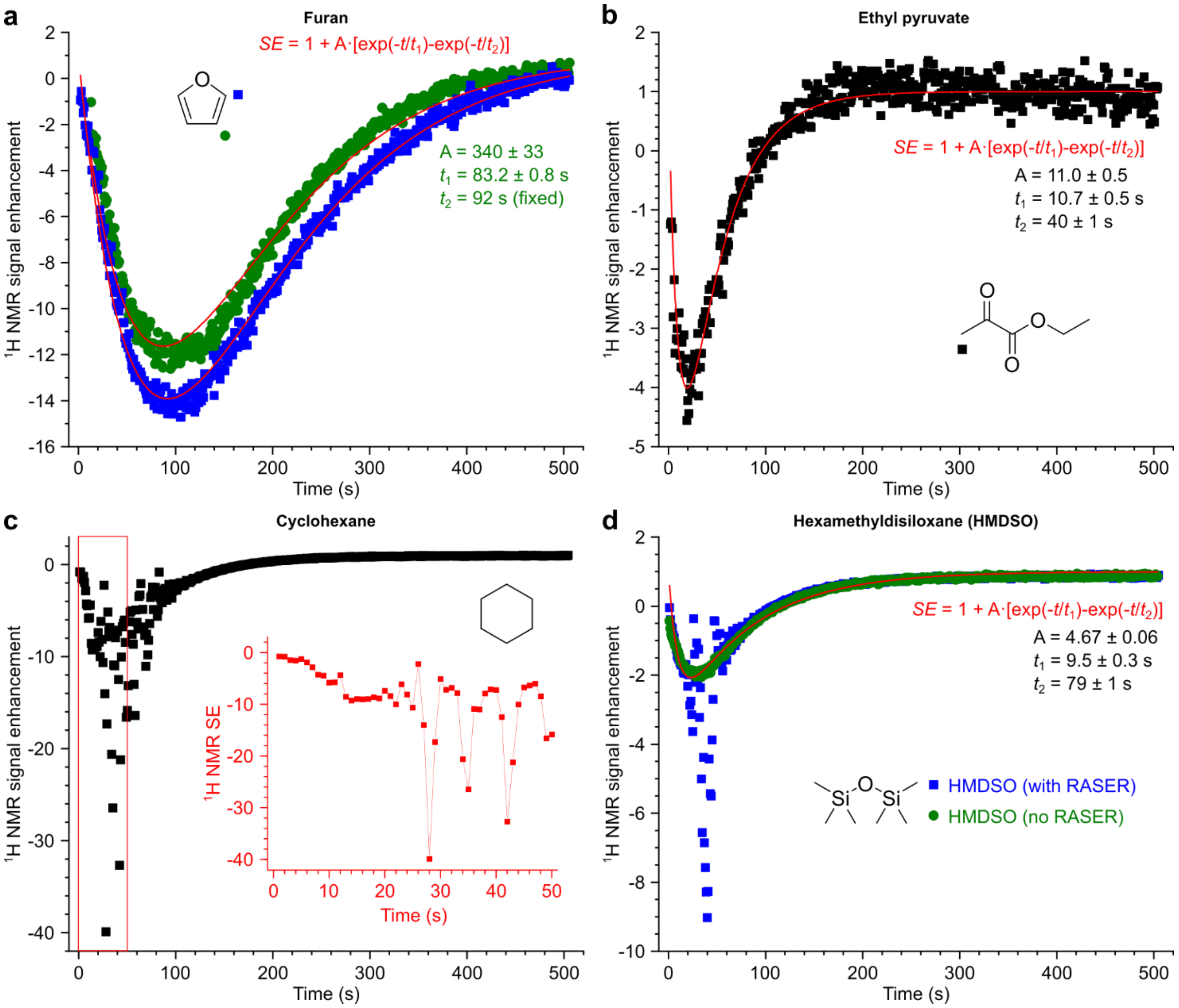

Figure S11. (**a**) Kinetics of PRINOE ^{1}H NMR signal enhancement for furan. Note that reliable estimation of fitting parameters for α-protons was not possible. (**b**) Kinetics of PRINOE ^{1}H NMR signal enhancement for the methyl group of pyruvate moiety in ethyl pyruvate. (**c**) Kinetics of PRINOE ^{1}H NMR signal enhancement for cyclohexane (with inset showing data acquired in the first 50 s). (**d**) Kinetics of PRINOE ^{1}H NMR signal enhancement for HMDSO.

Table S7. Average chemical conversion of **1'** to **1** (X), initial molar polarization of HP donor **1** and solutes (mP), fraction of achieved molar polarization of the solutes and HP donor **1** (|mP(solute)/mP(**1**)|), maximum ^{1}H NMR signal enhancement factors of solutes (SE), maximum normalized signal enhancements (SE/mP), and T_1 relaxation times for different solutes. T_1 values were measured at 7.05 T using an inversion-recovery protocol under H_2 pressure after hydrogenation.

Solute	***X*, %**	***mP*(1), *mM***	***mP*(solute), mM**	**|*mP*(solute)/*mP*(1)|**	***SE*(solute)**	***SE/mP*, M^{-1}**	**T_1, s**
Benzene	64 ± 4	101 ± 1	–0.82±0.20	≈8.1·10^{-3}	–42 ± 11	–420 ± 105	56 ± 1
Furan[a]	60 ± 3	116 ± 9	–0.27±0.01	≈2.3·10^{-3}	–13.8 ± 0.8	–120 ± 14	92 ± 6[60]
EtPyr[b]	31 ± 3	66 ± 6	–0.07±0.01	≈1.0·10^{-3}	–3.5 ± 0.6	–53 ± 6	–
CyH	56 ± 10	108 ± 6	–0.52±0.54	≈4.8·10^{-3}	–27 ± 28	–242 ± 240	18.4 ± 0.6
HMDSO[c]	62 ± 15	104 ± 14	–0.12±0.04	≈1.1·10^{-3}	–6 ± 2	–60 ± 21	10.0 ± 0.4
HMDSO[d]	59 ± 8	87 ± 3	–0.036±0.002	≈4·10^{-4}	–1.87 ± 0.09	–21.5 ± 0.8	

[a] Data for protons in the α-position. [b] Data for the methyl group of the pyruvate fragment. [c] Data from the spectra acquired in the experiments with ^{1}H RASER induced on HMDSO. [d] Data from the spectra acquired in the experiments without ^{1}H RASER induced on HMDSO.

Table S8. ^{1}H NMR spectrographs for CyH and HMDSO obtained after application of a single 6° RF pulse after the sample spent 59 s (for CyH) or 48 s (for HMDSO) inside the NMR probe.

**Target**	**RASER spectrographs**
CyH	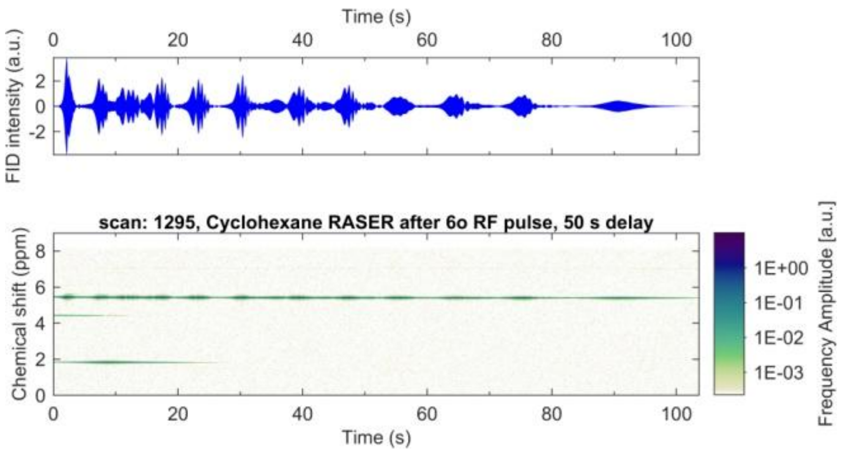

HMDSO	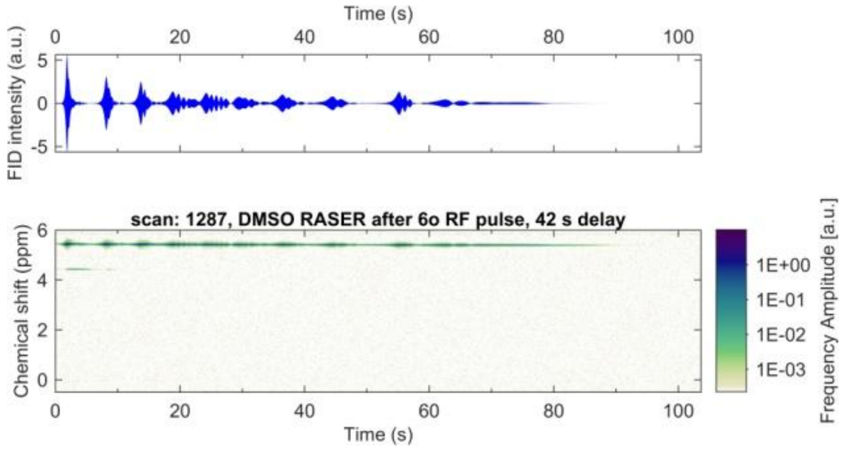

Table S9. ^{1}H NMR spectrographs for CyH and HMDSO obtained after application of a train of 1°/1 s RF pulses. 44 RF pulses were applied onto the sample with CyH, 36 RF pulses were applied onto the sample with HMDSO.

Target	RASER acquisition after a pulse train
CyH	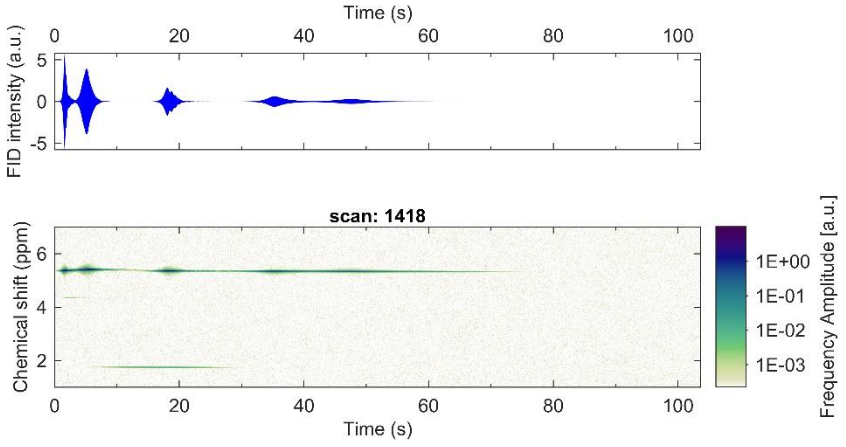
HMDSO	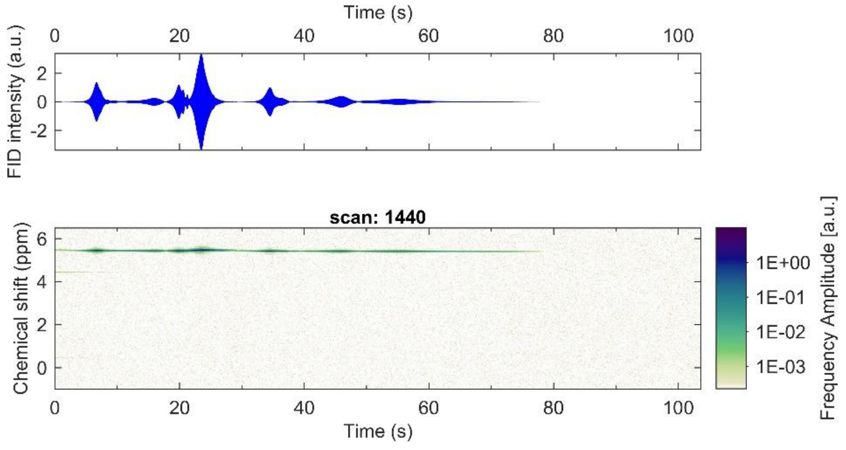

Section S10. PRINOE experiments at 1.4 T benchtop NMR spectrometer

The example PRINOE kinetics curve obtained for benzene at 1.4 T NMR spectrometer is shown in Figure S12. Note that conversion of **1** at these conditions was 95 ± 8%, while its molar polarization was only 68 ± 39 mM. The difference in hydrogenation kinetics and polarization efficiency between 7.05 and 1.4 T experiments can be attributed to the (at first glance, minor) modifications of the experimental setup. At 1.4 T setup a thinner 0.6 mm OD gas supply catheter was used vs. 1.6 mm (1/16'') OD catheter at 7.05 T setup, potentially leading to more efficient gas/liquid mixing and more efficient hydrogenation.

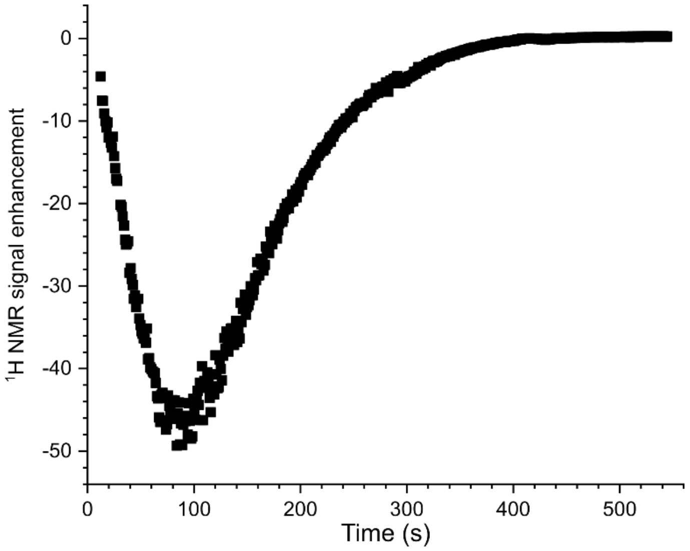

Figure S12. Kinetics of PRINOE ^{1}H NMR signal enhancement for benzene at 1.4 T benchtop NMR spectrometer.

References

[1] N. V. Chukanov, O. G. Salnikov, R. V. Shchepin, K. V. Kovtunov, I. V. Koptyug, E. Y. Chekmenev, "Synthesis of Unsaturated Precursors for Parahydrogen-Induced Polarization and Molecular Imaging of 1-^{13}C-Acetates and 1-^{13}C-Pyruvates via Side Arm Hydrogenation" *ACS Omega* **2018**, *3*, 6673–6682.

[2] I. A. Trofimov, O. G. Salnikov, A. N. Pravdivtsev, H. De Maissin, A. P. Yi, E. Y. Chekmenev, J.-B. Hövener, A. B. Schmidt, I. V. Koptyug, "Through-bond and through-space radiofrequency amplification by stimulated emission of radiation" *Commun. Chem.* **2024**, *7*, 235.

[3] X. Mao, C. Ye, "Line shapes of strongly radiation-damped nuclear magnetic resonance signals" *J. Chem. Phys.* **1993**, *99*, 7455–7462.

[4] J.-H. Chen, B. Cutting, G. Bodenhausen, "Measurement of radiation damping rate constants in nuclear magnetic resonance by inversion recovery and automated compensation of selective pulses" *J. Chem. Phys.* **2000**, *112*, 6511–6514.

[5] I. Solomon, "Relaxation Processes in a System of Two Spins" *Phys. Rev.* **1955**, *99*, 559–565.

[6] S. Appelt, A. Kentner, S. Lehmkuhl, B. Blümich, "From LASER physics to the *para*-hydrogen pumped RASER" *Prog. Nucl. Magn. Reson. Spectrosc.* **2019**, *114–115*, 1–32.

[7] H.-Y. Chen, Y. Lee, S. Bowen, C. Hilty, "Spontaneous emission of NMR signals in hyperpolarized proton spin systems" *J. Magn. Reson.* **2011**, *208*, 204–209.

[8] S. Macura, R. R. Ernst, "Elucidation of cross relaxation in liquids by two-dimensional N.M.R. spectroscopy" *Mol. Phys.* **1980**, *41*, 95–117.

[9] A. Abragam, *The principles of nuclear magnetism*, Oxford Univ. Pr, Oxford, **2011**.

[10] A. S. Kiryutin, K. L. Ivanov, A. V. Yurkovskaya, R. Kaptein, H. M. Vieth, "Transfer of parahydrogen induced polarization in scalar coupled systems at variable magnetic field" *Z. Phys. Chem.* **2012**, *226*, 1343–1362.

[11] C. R. Bowers, D. P. Weitekamp, "Transformation of Symmetrization Order to Nuclear-Spin Magnetization by Chemical Reaction and Nuclear Magnetic Resonance" *Phys. Rev. Lett.* **1986**, *57*, 2645–2648.

[12] O. G. Salnikov, I. A. Trofimov, A. N. Pravdivtsev, K. Them, J.-B. Hövener, E. Y. Chekmenev, I. V. Koptyug, "Through-Space Multinuclear Magnetic Resonance Signal Enhancement Induced by Parahydrogen and Radiofrequency Amplification by Stimulated Emission of Radiation" *Anal. Chem.* **2022**, *94*, 15010–15017.
